# Supplementary material for: The ubiquitin ligase Pellino1 targets STAT3 to regulate macrophage-mediated inflammation and tumor development
Source: Nat Commun. 2025 Feb 1;16:1256. doi: 10.1038/s41467-025-56440-6 (PMC11787384; doi:10.1038/s41467-025-56440-6)

Supplementary Information

The ubiquitin ligase Pellino1 targets STAT3 to regulate macrophage-mediated inflammation and tumor development

(Running title: Receptor-signal responsive ubiquitination orchestrates the development of colitis-associated cancer)

Soeun Hwang[1], Junhee Park[1], Seo-Young Koo[1], Si-Yeon Lee[1], Yunju Jo[2], Dongryeol Ryu[2], Heounjeong Go[3], and Chang-Woo Lee[1,4,*]

[1]Department of Molecular Cell Biology, Samsung Medical Center, Sungkyunkwan University School of Medicine, Suwon 16419, South Korea, [2]Department of Biomedical Science and Engineering, Gwangju Institute of Science and Technology (GIST), Gwangju 61005, South Korea, [3]Department of Pathology, University of Ulsan College of Medicine, Asan Medical Center, Seoul 05505, South Korea, [4]Research Institute, Curogen Technology, Suwon 16419, South Korea

Supplementary Figures 1-16
Supplementary Table 1
Uncropped gels and blots for Supplementary Figures

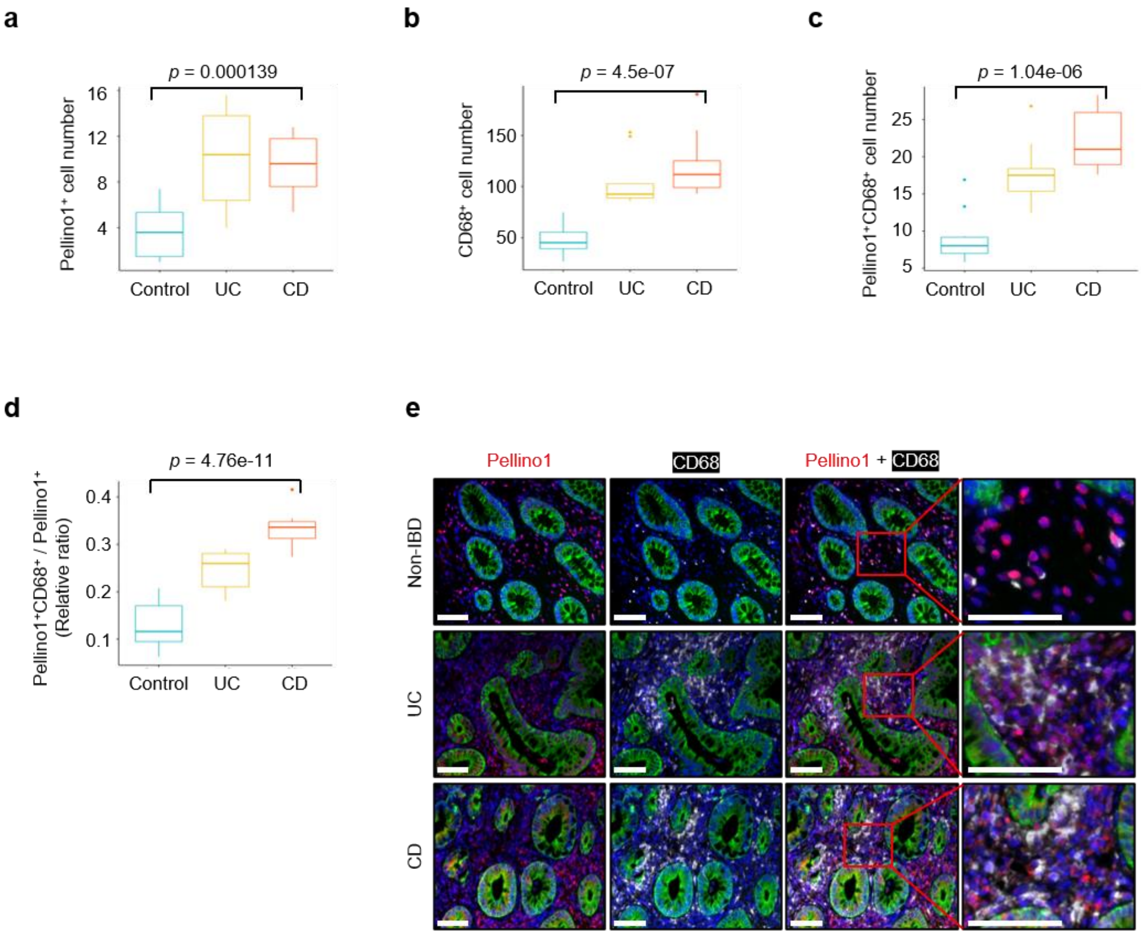

Supplementary Fig. 1. Increased Pellino1 expression in inflamed intestinal tissues from patients with IBD. a Numbers of cells expressing Pellino1 in colonic epithelia of healthy controls (n = 10), UC (n = 11), and CD patients (n = 11). **b** Numbers of cells expressing CD68 in colonic epithelia and lamina propria of healthy controls (n = 10), UC (n = 11), and CD patients (n = 11). **c** Numbers of cells co-expressing Pellino1 and CD68 in colonic epithelia and lamina propria of healthy controls (n = 10), UC (n = 11), and CD patients (n = 11). **d** Relative ratios of Pellino1 in colonic epithelia and lamina propria of healthy controls (n = 10), UC (n = 11), and CD patients (n = 11). **e** Representative immunofluorescence staining of Pellino1 (red), CD68 (white), cytokeratin (green), and DAPI (blue) in colonic tissues from healthy individuals, UC, and CD patients. DAPI was used to stain the nuclei. Scale bar = 50 µm. Data are presented as mean ± SEM. Statistical comparisons were performed using one-way ANOVA. Source data are provided as a Source Data file.

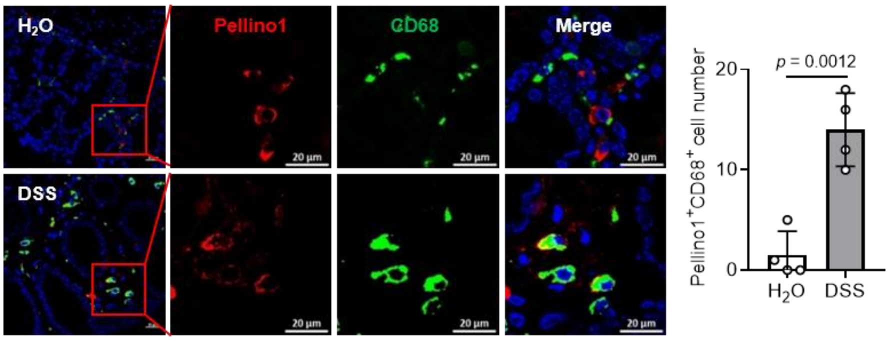

Supplementary Fig. 2. Enhanced Expression of macrophage Pellino1 in colitis. (Left) Immunofluorescence staining of Pellino1 (red), CD68 (green), and DAPI (blue) in colon tissues of WT male mice drinking normal or 1.5% DSS water for 9 days. DAPI was used to stain the nuclei. Scale bar = 20 μm. (Right) Number of Pellino1^{+}CD68^{+} cells (n = 4). Quantification of cells was performed in the field of view area. Data are presented as mean ± SD. Statistical comparisons were made using two-tailed Student's t test. Source data are provided as a Source Data file.

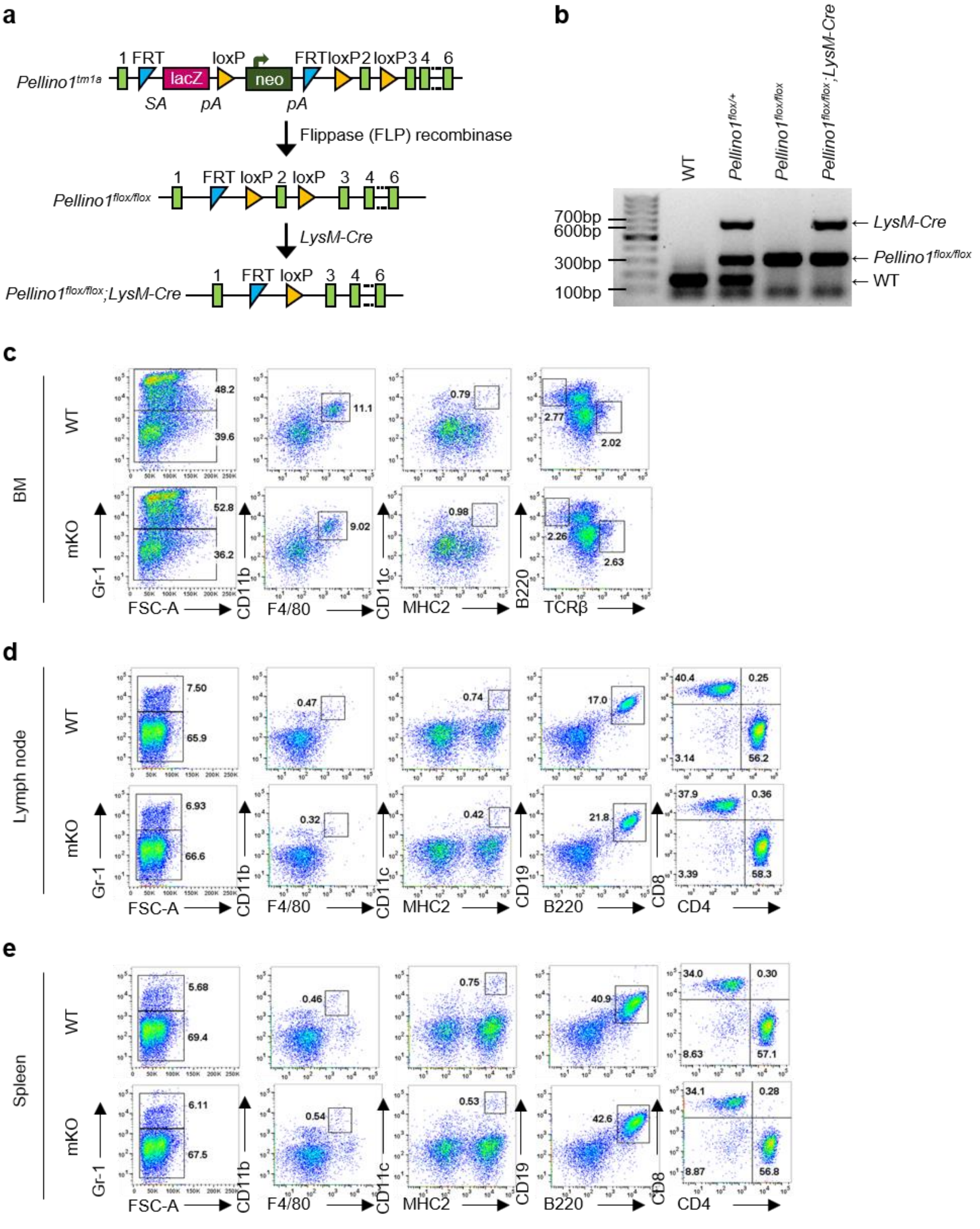

Supplementary Fig. 3. Generation of a macrophage-specific Pellino1 deletion mouse model. a Targeting strategy for *Pellino1*$^{flox/flox}$*; LysM-Cre* mice. *Pellino1*$^{flox/flox}$*; LysM-Cre* mice were generated by crossing *Pellino1*$^{flox/flox}$ mice with those expressing *LysM-Cre* recombinase, resulting in recombination at loxP sites and subsequent deletion of both the neomycin cassette and exon 2. **b** PCR genotyping assay of WT, *Pellino1*$^{flox/+}$, *Pellino1*$^{flox/flox}$, and *Pellino1*$^{flox/flox}$*; LysM-Cre* mice. **c** Representative flow cytometric analysis showing neutrophils ($Gr1^{+}$),

macrophages ($CD11b^{+}F4/80^{+}$), dendritic cells ($CD11c^{+}MHC^{+}$), B cells ($B220^{+}$), and T cells ($TCR\beta^{+}$) gated on $CD45^{+}$ from BM of 8-week-old WT or Pellino1-mKO male mice. **d**, **e** Representative flow cytometric analysis showing neutrophils ($Gr1^{+}$), macrophages ($CD11b^{+}F4/80^{+}$), dendritic cells ($CD11c^{+}MHC^{+}$), B cells ($CD19^{+}B220^{+}$), and T cells subsets ($CD4^{+}CD8^{-}$/$CD4^{-}CD8^{+}$) gated on $CD45^{+}$ from lymph nodes and spleens of WT or Pellino1-mKO male mice. Source data are provided as a Source Data file.

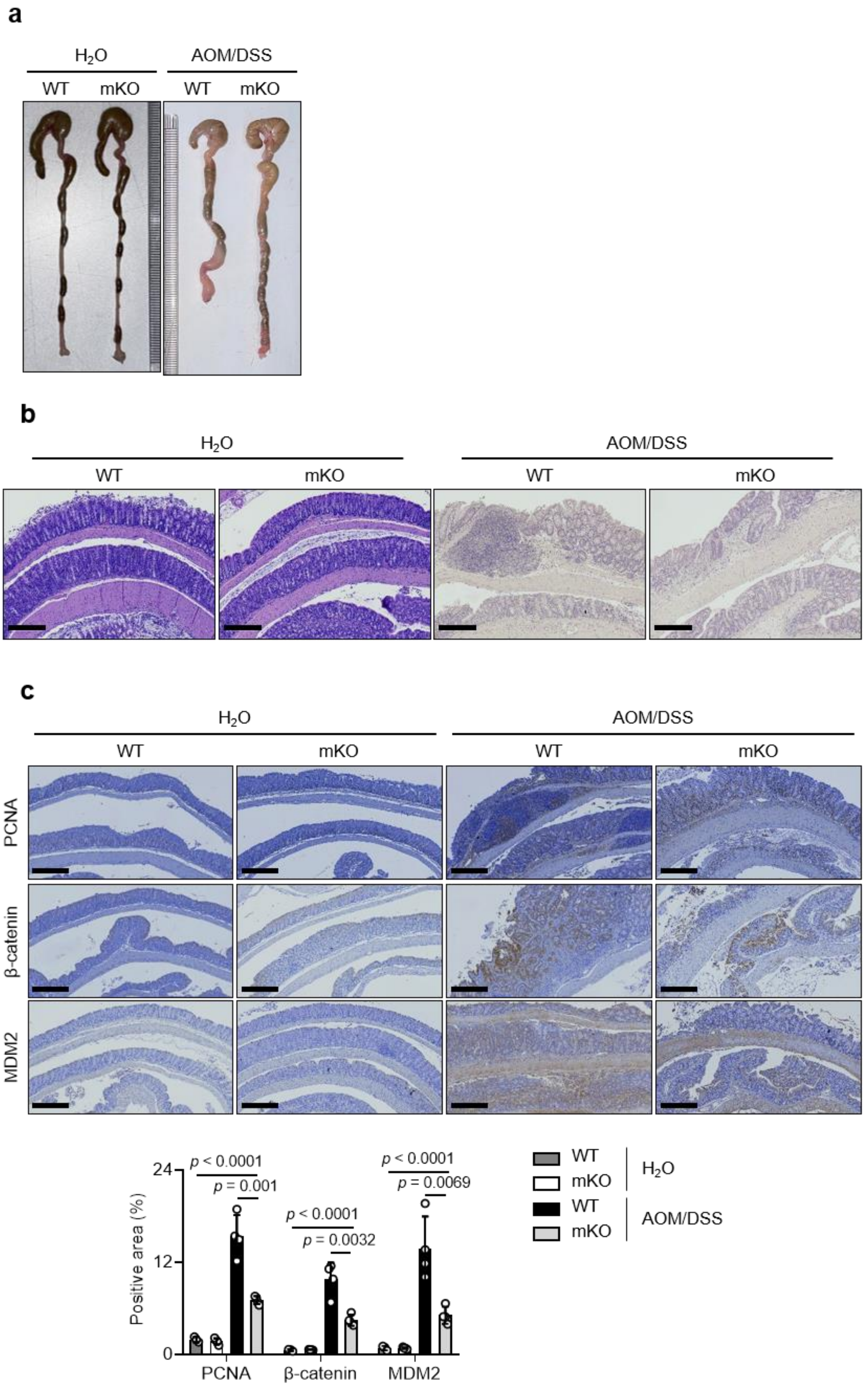

Supplementary Fig. 4. Development and validation of the AOM/DSS-induced CAC model.
a Macroscopic images of colons from WT and Pellino1-mKO male mice in normal or

AOM/DSS conditions collected at 20 weeks. **b** Histological images of colons from WT and Pellino1-mKO male mice under normal or AOM/DSS conditions collected at 20 weeks. Scale bar = 300 µm. **c** (Top) Representative immunohistochemical staining images for PCNA, β-catenin, and MDM2 in colon tissues of WT and Pellino1-mKO male mice under normal or AOM/DSS conditions. Scale bar = 300 µm. (Bottom) Quantification of the positive area for PCNA, β-Catenin, and MDM2. Measurement of area within the field of view (H_2O-WT, *n* = 3; H_2O-Pellino1-mKO, *n* = 4; AOM/DSS-WT, *n* = 4; AOM/DSS-Pellino1-mKO, *n* = 4). Data are presented as mean ± SD. Statistical comparisons were made using two-tailed Student's t test and one-way ANOVA. Source data are provided as a Source Data file.

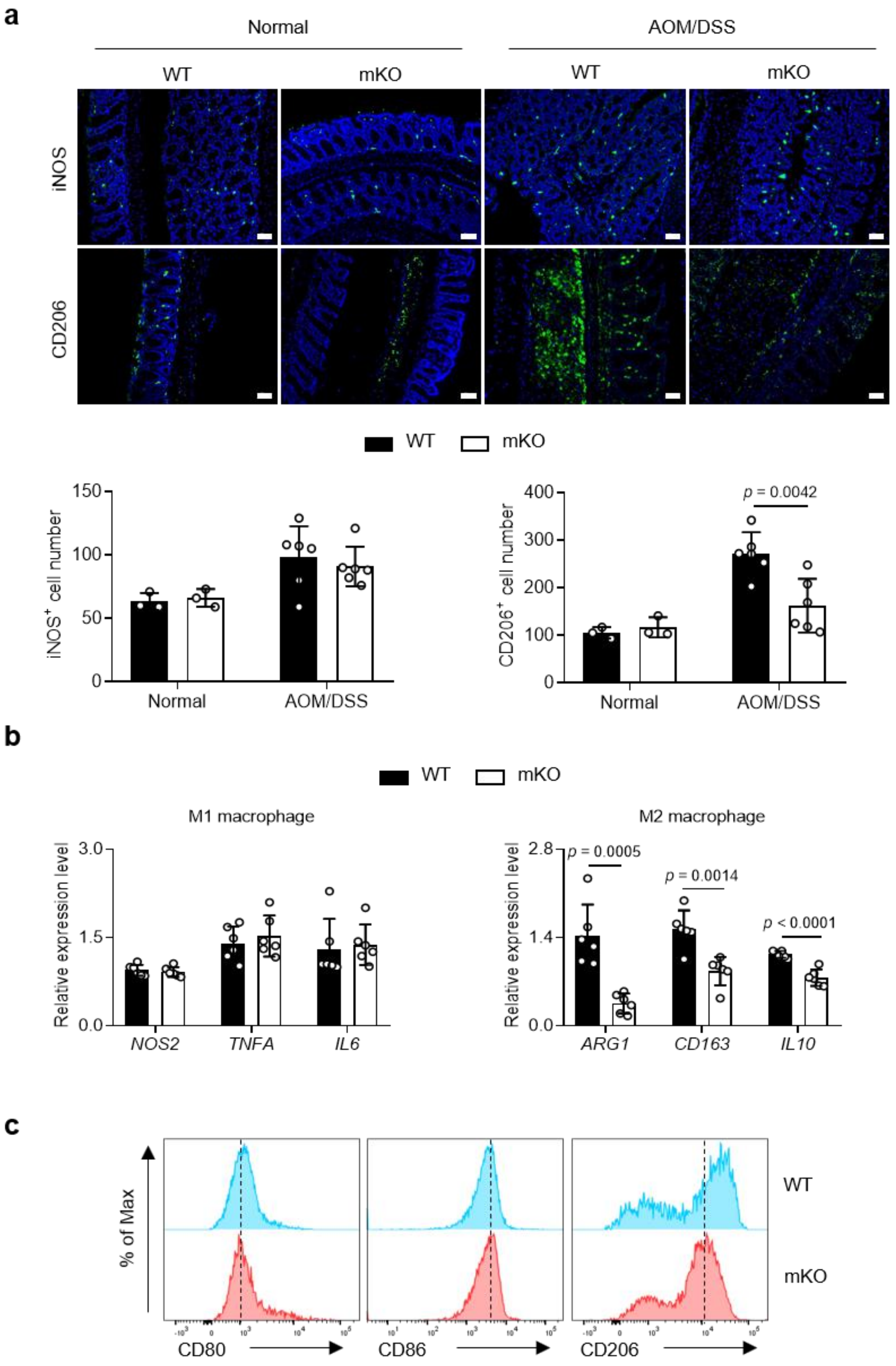

Supplementary Fig. 5. Pellino1 deficiency reduces M2 cells in colons of AOM/DSS-induced CAC mice. a (Top) Immunofluorescence staining performed on iNOS^{+} (M1 macrophage marker) and CD206^{+} (M2 macrophage marker) colonic macrophages of WT and Pellino1-mKO male mice following AOM/DSS challenge. Scale bar = 50 µm. (Bottom)

Quantification of $iNOS^+$ or $CD206^+$ cells using ImageJ (Normal, $n = 3$; AOM/DSS, $n = 6$). Quantification of cells was performed in the field of view area. **b** mRNA expression levels of M1 macrophage markers (*NOS2, TNFA, IL6*) and M2 macrophage markers (*ARG1, CD163, IL10*) in intestinal macrophages from WT and Pellino1-mKO male mice with AOM/DSS-induced CAC were analyzed ($n = 6$). **c** A flow cytometric histogram of M1 markers (CD80 and CD86) and an M2 marker (CD206) in intestinal $CD45^+$ cells of WT and Pellino1-mKO male mice with AOM/DSS-induced CAC. Representative overlay histograms showing mean fluorescence intensity (MFI). Data were represented as mean ± SD in **a**, **b**. All statistical comparisons were made using two-tailed Student's t test. Source data are provided as a Source Data file.

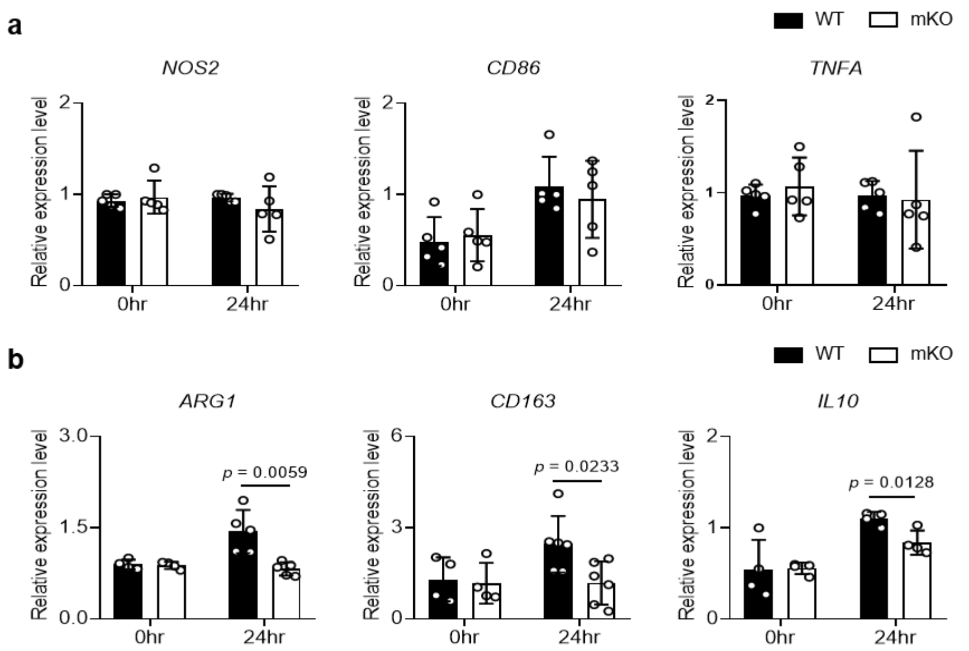

Supplementary Fig. 6. Pellino1 promotes M2 macrophage polarization. a qRT-PCR analysis of *NOS2*, *CD86*, and *TNFA* mRNA levels in WT and Pellino1-mKO BMDMs after polarization. Cells were polarized to M1 macrophages by treatment with 100 ng/ml LPS and 20 ng/ml IFN γ for 24 hours. The mRNA level was normalized with GAPDH (n = 5). **b** qRT-PCR analyses of *ARG1* (0hr, n = 4; 24hr, n = 5), *CD163* (0hr, n = 4; 24hr, n = 6), and *IL10* (n = 4) mRNA levels in WT and Pellino1-mKO BMDMs after polarization. Cells were polarized to M2 macrophages by treatment with 20 ng/ml IL-4 and 20 ng/ml IL-13 for 24 hours. The mRNA level was normalized with GAPDH. Data were represented as mean ± SD in **a**, **b**. All statistical comparisons were made using two-tailed Student's t test. Source data are provided as a Source Data file.

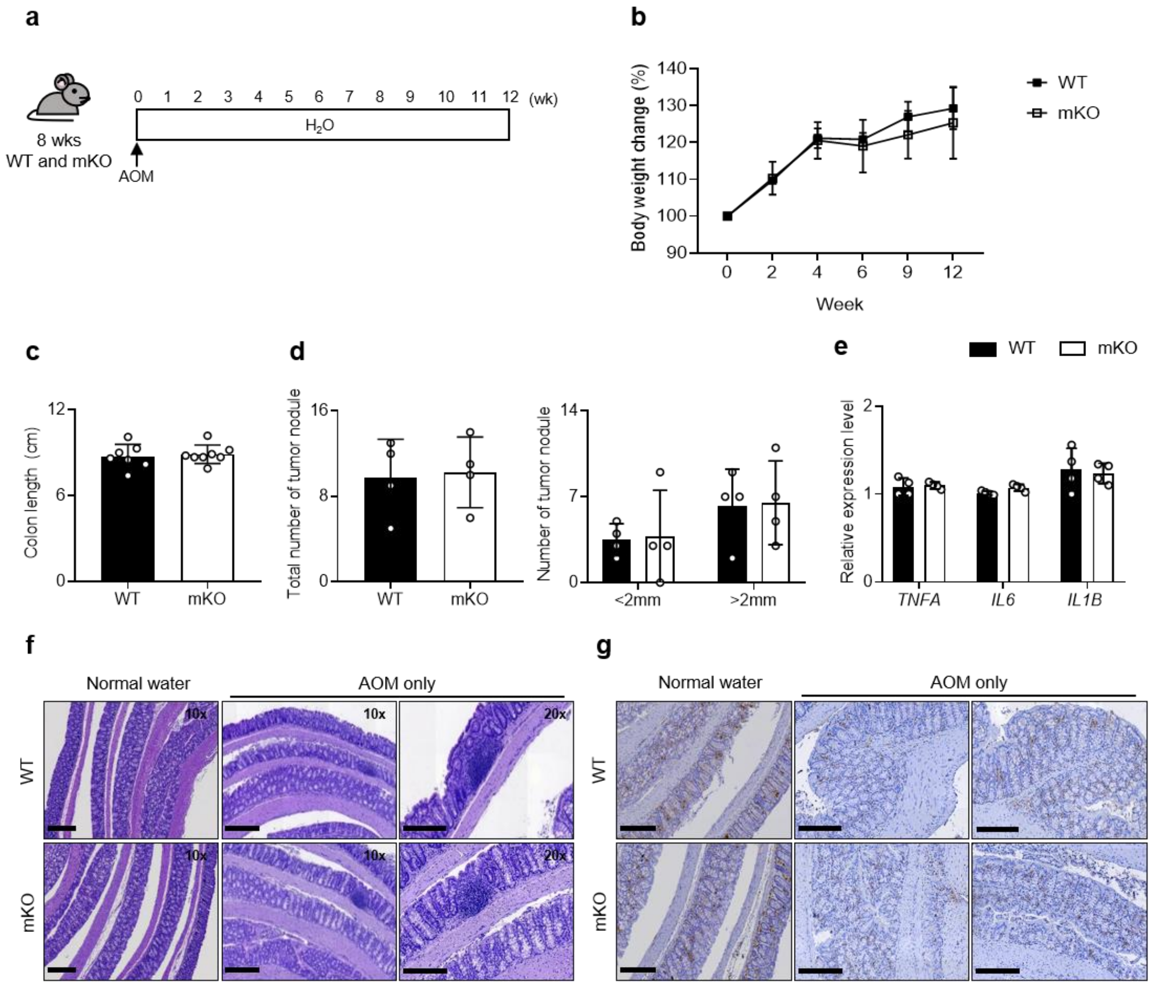

Supplementary Fig. 7. Effect of only AOM on WT and Pellino1-mKO male mice. a Protocol for inducing colon cancer with AOM treatment. WT and Pellino1-mKO male mice received a single intraperitoneal injection of AOM (10 mg/kg). Mice were euthanized on day 84 after AOM administration for tumor formation analysis. **b** Body weight was monitored for AOM-induced CAC mice (n = 4). **c** Measurement of colon length at the time of euthanization on day 84 after AOM administration (n = 4). **d** (Left) Total number of tumor nodules in the entire colon. (Right) Numbers of small tumors (< 2 mm) and large tumors (> 2 mm) in the entire colon (n = 4). **e** Levels of *TNFA*, *IL6*, and *IL1B* mRNAs in colon tissues of WT and Pellino1-mKO male mice after AOM administration were measured with qRT-PCR and normalized to GAPDH expression (n = 4). **f** H&E staining was performed for colon tissue sections of mice drinking normal water and mice subjected to AOM challenge. Scale bar = 300 μm for 10x sections and 200 μm for 20x sections. **g** Immunohistochemical staining for macrophage marker F4/80 was performed using colonic tissue sections from mice drinking normal water and mice subjected to AOM challenge. Scale bar = 200 μm. Data were

represented as mean ± SD in **b**, **c**, **d**, **e**. Source data are provided as a Source Data file.

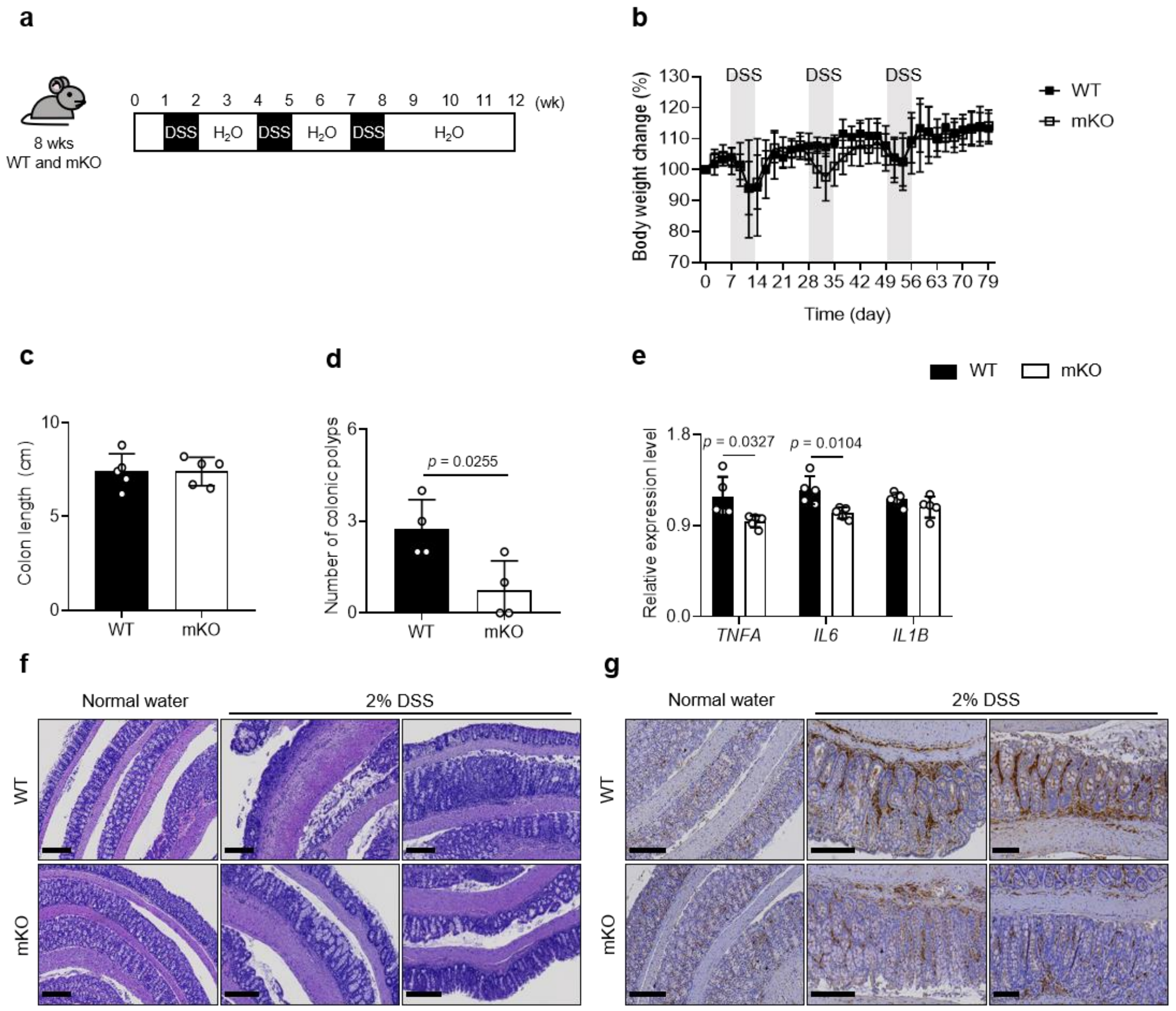

Supplementary Fig. 8. Effect of 2% chronic DSS on WT and Pellino1-mKO male mice. a Protocol for inducing chronic colitis with DSS administration. WT and Pellino1-mKO male mice were subjected to three cycles of 2% DSS administration. **b** Body weight was monitored for DSS-induced chronic colitis mice ($n = 5$). **c** Measurement of colon length at the time of euthanization on day 84 after starting the experiment ($n = 5$). **d** Measurement of the number of colonic polyps in the entire colon ($n = 4$). **e** Expression levels of *TNFA*, *IL6*, and *IL1B* mRNA in colon tissues of WT and Pellino1-mKO male mice after 2% DSS administration were measured by qRT-PCR and normalized to GAPDH expression ($n = 5$). **f** H&E staining of colonic tissue sections from mice drinking normal water and mice with 2% DSS-induced chronic colitis. Scale bar = 300 μm. **g** Immunohistochemical staining for the macrophage marker F4/80 was performed using colonic tissue sections from mice drinking normal water and mice with 2% DSS-induced chronic colitis. Scale bar = 200 μm. Data were represented as mean ± SD in **b**, **c**, **d**, **e**. All statistical comparisons were made using two-tailed Student's t test.

Source data are provided as a Source Data file.

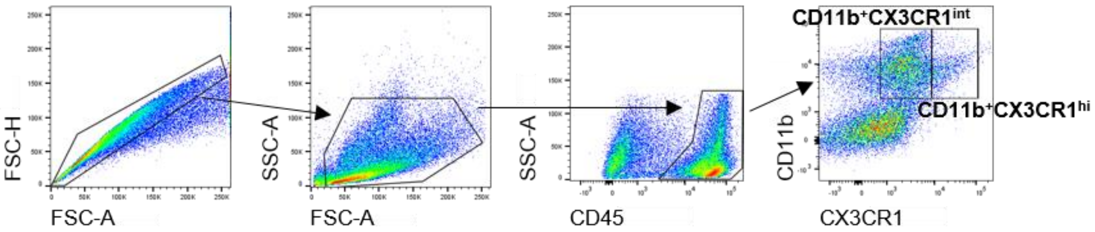

Supplementary Fig. 9. Gating strategies used for flow cytometric analysis. Gating strategy to identify migratory macrophages (CD45$^+$CD11b$^+$CX3CR1^{int}) and resident macrophages (CD45$^+$CD11b$^+$CX3CR1^{hi}) in the intestinal lamina propria, as presented in Fig. 5a, b.

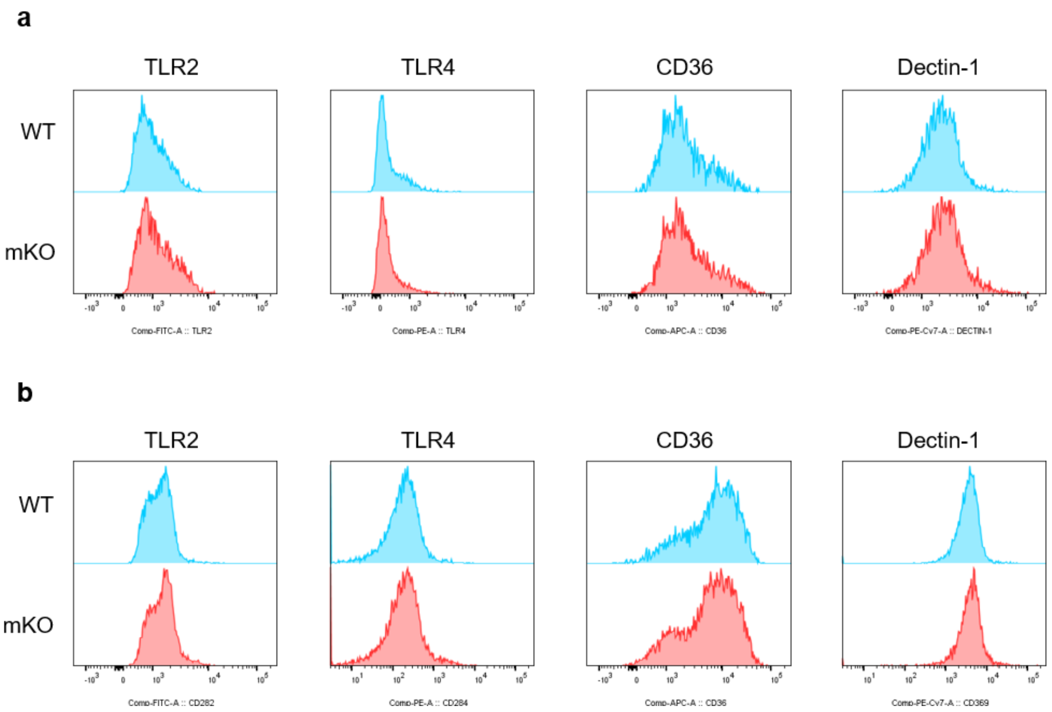

Supplementary Fig. 10. Pellino1 deficiency does not affect antigen recognition by macrophages. a A flow cytometric histogram of TLR2, TLR4, CD36, and Dectin-1 expression in F4/80$^+$CD11B$^+$ cells of WT and Pellino1-mKO mice of normal and **b** AOM/DSS groups. TLR2, TLR4, CD36, and Dectin-1 expression levels on F4/80$^+$CD11B$^+$ cells were analyzed by flow cytometry using FITC-conjugated mouse anti-TLR2, PE-conjugated mouse anti-TLR4, APC-coupled hamster anti-CD36, and PE-Cyanine7 coupled mouse anti-CD369 (Dectin-1). Histograms represent data from four independent experiments. Source data are provided as a Source Data file.

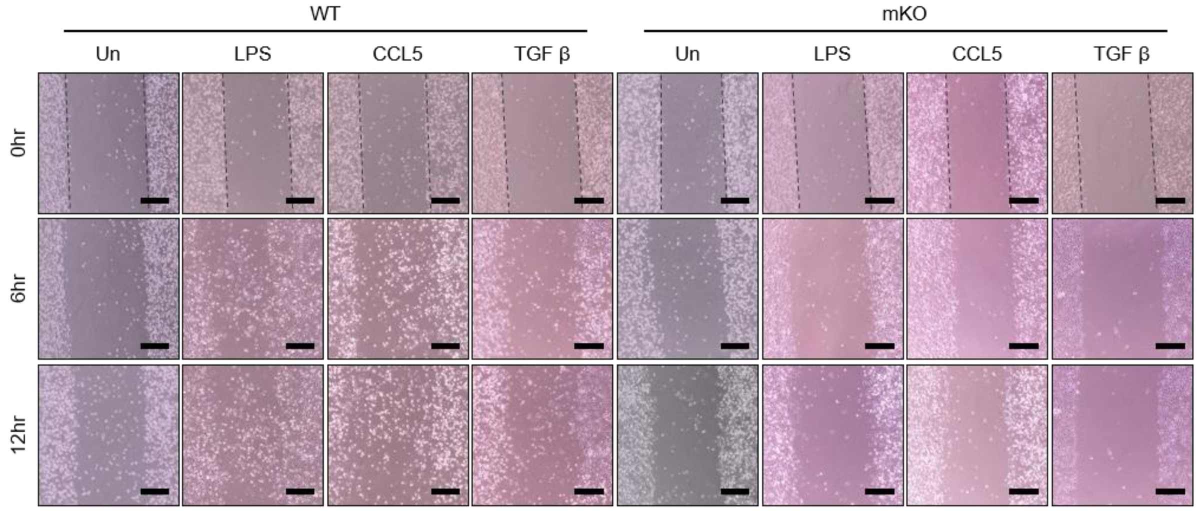

Supplementary Fig. 11. Reduced macrophage migration in Pellino1-mKO mice. Representative images illustrate wound healing analysis in BMDMs derived from WT and Pellino1-mKO mice. Cells were treated with 100 ng/mL LPS, 20 ng/mL CCL5, and 100 ng/mL TGF β at intervals of 6 hours and 12 hours. Initial wounded areas were marked with a dashed line. Scale bar = 100 μm.

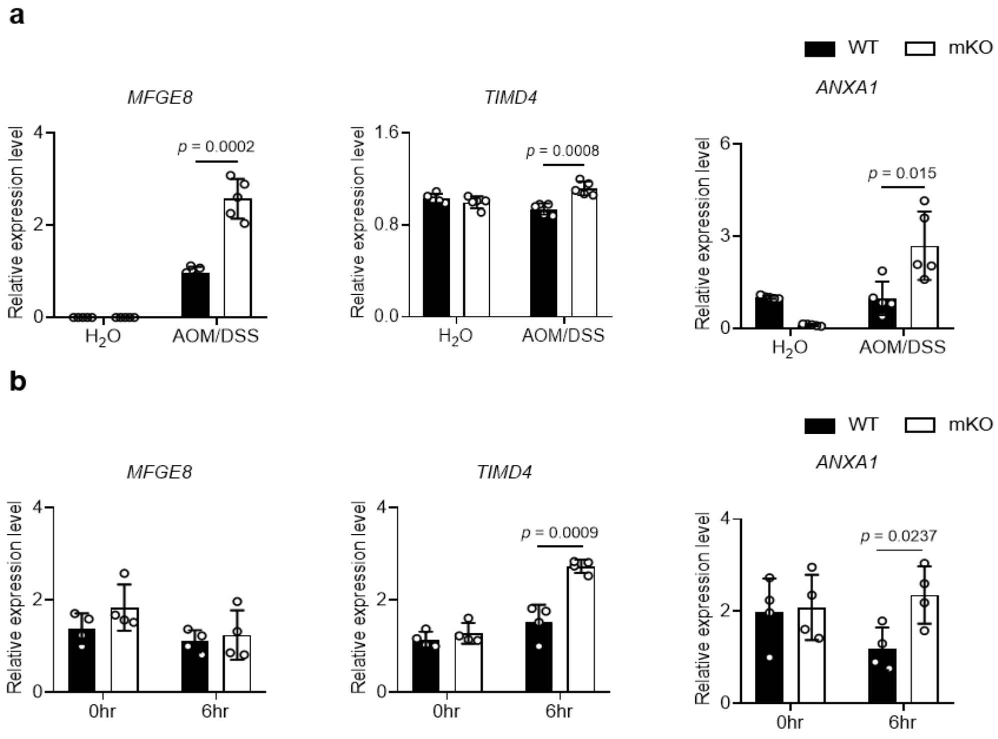

Supplementary Fig. 12. Pellino1 deficiency enhances phagocytic activity in macrophages. **a, b** mRNA expression levels of phagocytosis-related genes in intestinal macrophages and BMDMs from WT and Pellino1-mKO mice were assessed by qRT-PCR and normalized to GAPDH expression. **a** F4/80$^+$ macrophages were isolated from colons of WT and Pellino1-mKO male mice with normal and AOM/DSS-induced CAC ($n = 5$). **b** BMDMs were stimulated with 100 ng/mL LPS for 6 hours ($n = 4$). Data were represented as mean ± SD in **a**, **b**. All statistical comparisons were made using two-tailed Student's t test. Source data are provided as a Source Data file.

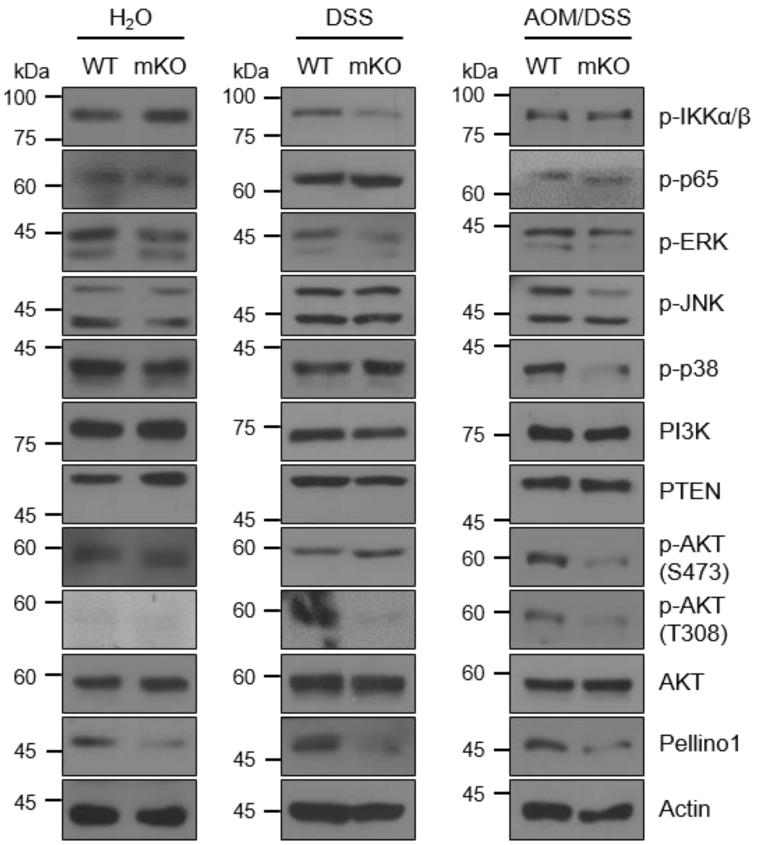

Supplementary Fig. 13. Pellino1 deficiency attenuates the activation of several protein kinases. Immunoblot analyses of intestinal macrophages isolated from WT and Pellino1-mKO male mice in normal, acute 1.5% DSS, and AOM/DSS groups were performed. Source data are provided as a Source Data file.

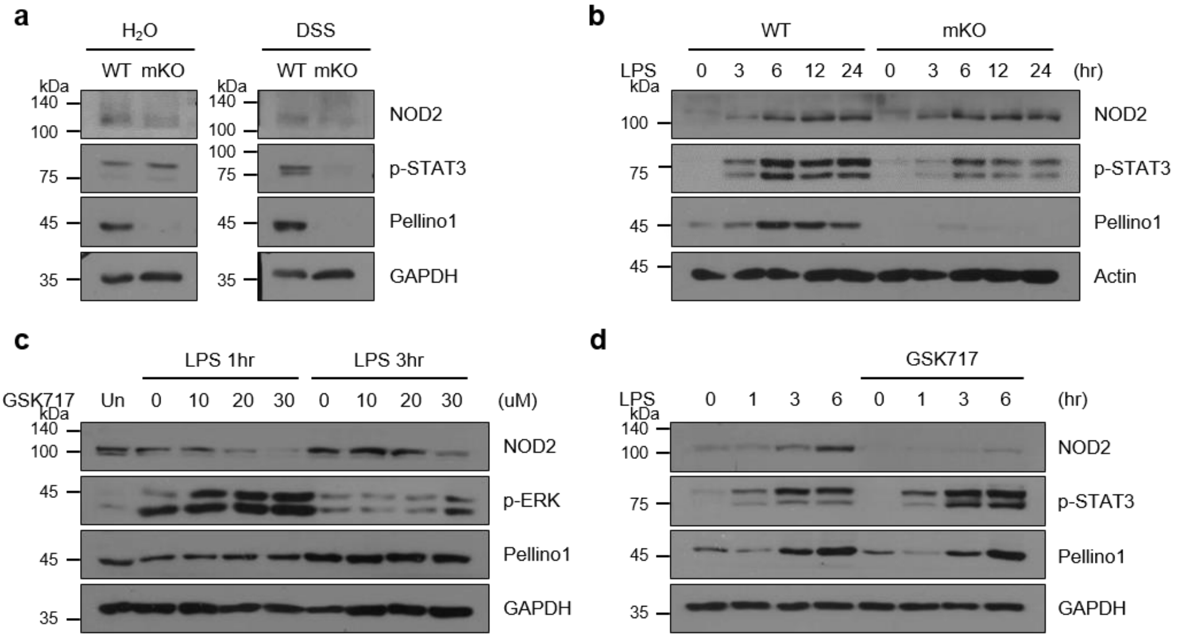

Supplementary Fig. 14. Analysis of NOD2 and Pellino1 in p-STAT3 regulation in macrophages. a Immunoblot analyses of Pellino1, p-STAT3, and NOD2 protein levels in lysates of intestinal macrophages isolated from WT and Pellino1-mKO male mice in normal and acute 1.5% DSS groups. GAPDH was used as a loading control. **b** Immunoblot analysis was performed to assess Pellino1, p-STAT3, and NOD2 protein levels in lysates of BMDMs from WT and Pellino1-mKO mice. BMDMs were stimulated with 100 ng/mL LPS for the indicated time. Actin was used as a loading control. **c** RAW cells were treated with a specific NOD2 inhibitor GSK717 for 2 hours at indicated concentration, followed by stimulation with 100 ng/mL LPS for 1 hour and 3 hours. Cell lysates were subjected to immunoblotting analysis using antibodies against NOD2, p-ERK, Pellino1, and GAPDH. GAPDH was used as a loading control. **d** WT BMDM cells were treated with 30 μM of the NOD2 inhibitor GSK717 for 2 hours, followed by stimulation with 100 ng/mL LPS for the indicated times. Cell lysates were subjected to immunoblotting analysis using antibodies against NOD2, p-STAT3 (Y705), Pellino1, and GAPDH. GAPDH was used as a loading control. Source data are provided as a Source Data file.

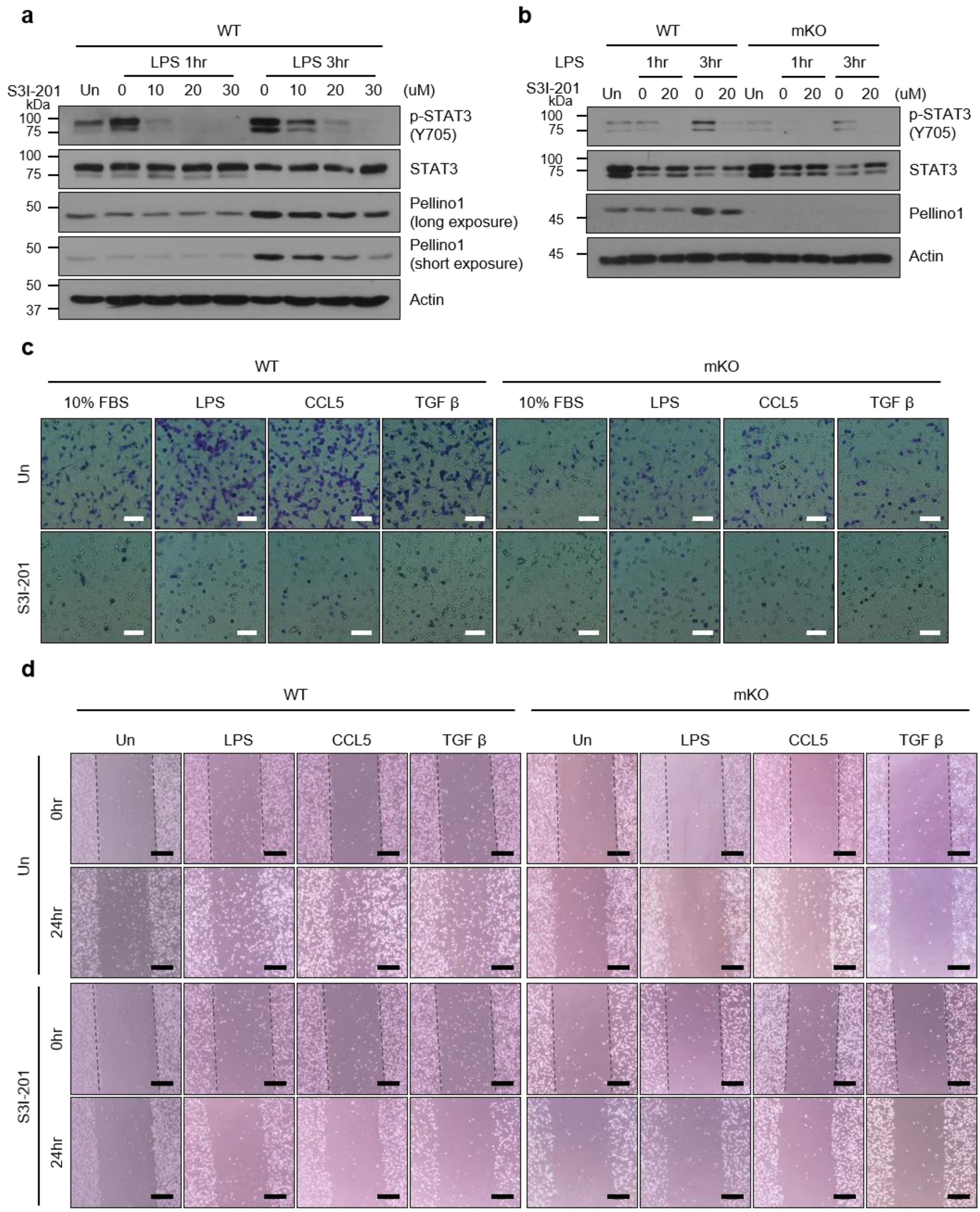

Supplementary Fig. 15. Effect of STAT3 on macrophage migration and its relationship with Pellino1. a WT BMDM cells were treated with a specific STAT3 inhibitor S3I-201 for 30 minutes at indicated concentration, followed by stimulation with 100 ng/mL LPS for 1 hour and 3 hours. Cell lysates were subjected to immunoblotting analysis using antibodies against p-STAT3 (Y705), STAT3, Pellino1, and Actin. Actin was used as a loading control. **b** WT and

Pellino1-mKO BMDMs were treated with 20 μM S3I-201 for 30 minutes, followed by stimulation with 100 ng/mL LPS for 1 hour and 3 hours. Cell lysates were subjected to immunoblotting analysis using antibodies against p-STAT3 (Y705), STAT3, Pellino1, and Actin. Actin was used as a loading control. **c** Representative images of the migration assay. WT and Pellino1-mKO BMDMs were treated with 20 μM S3I-201 for 30 minutes and stimulated with 100 ng/mL LPS, 20 ng/mL CCL5, or 100 ng/mL TGF β for 24 hours. Scale bar = 50 μm. **d** Representative images of the wound healing assay. WT and Pellino1-mKO BMDMs were treated with 20μM S3I-201 for 30 minutes and stimulated with 100 ng/mL LPS, 20 ng/mL CCL5, or 100 ng/mL TGF β for 24 hours. Initial wounded areas were marked with a dashed line. Scale bar = 100 μm. Source data are provided as a Source Data file.

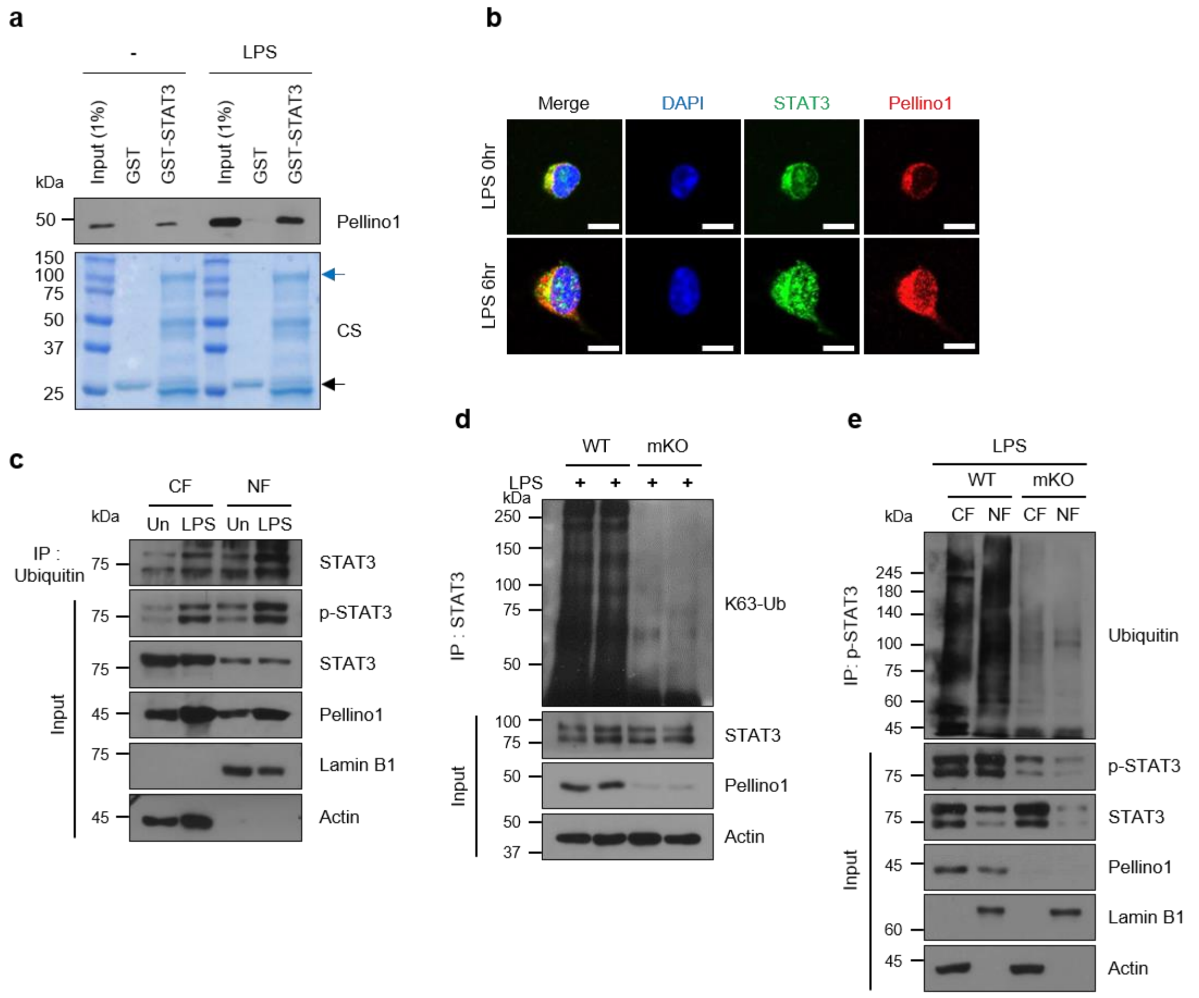

Supplementary Fig. 16. Pellino1 interacts with STAT3 and mediates its ubiquitination. a GST pulldown assays were performed using WT BMDMs. Cells were stimulated with 100 ng/mL LPS for 6 hours. Lysates were incubated with GST or GST-STAT3. Bound proteins were immunoblotted with an anti-Pellino1 antibody. GST, black arrow; GST-STAT3, blue arrow; CS, Coomassie brilliant blue staining. **b** Co-localization of Pellino1 with STAT3 in WT BMDMs. Immunofluorescence staining results of Pellino1 (red), STAT3 (green), and DAPI (blue) are shown. DAPI was used to stain the nuclei. Scale bar = 10 μm. **c** RAW cells were fractionated after 6 hours of treatment with 100 ng/mL LPS. Cell lysates were fractionated according to the protocol described in the Methods section. Each of the cellular and nuclear extracts was immunoprecipitated using an anti-Ubiquitin antibody and subsequently immunoblotted with an anti-STAT3 antibody. CF, cytosolic fraction; NF, nuclear fraction. **d** Immunoblot analysis of K63 ubiquitination of STAT3 in LPS-stimulated WT and Pellino1-mKO BMDMs. WT and Pellino1-mKO BMDMs were stimulated with 100 ng/mL LPS for 6 hours. BMDM lysates

were then immunoprecipitated with an anti-STAT3 antibody and immunoblotted with an anti-K63-Ub antibody. **e** WT and Pellino1-mKO BMDMs were fractionated after 3 hours of treatment with 100 ng/mL LPS. Cell lysates were fractionated according to the protocol described in the Methods section. Cell lysates were then immunoprecipitated with an anti-p-STAT3 antibody and immunoblotted with an anti-Ub antibody. CF, cytosolic fraction; NF, nuclear fraction. Source data are provided as a Source Data file.

Supplementary Table 1. List of primers used for qRT-PCR

**Mouse gene**	**Forward Primer**	**Reverse Primer**
Pellino1	5’-TCAGGCTGCCAAGGCAATAA-3’	5’-GGACTTTCAGTTGACCGACCA-3’
IL1B	5’-GAAATGCCACCTTTTGACAGTG-3’	5’-TGGATGCTCTCATCAGGACAG-3’
16s rRNA	5’-GTGGTGCATGGTTGTCGTCA-3’	5’-ACGTCGTCCCCACCTTCCTC-3’
TNFA	5’-CCTGTAGCCCACGTCGTAG-3’	5’-GGGAGTAGACAAGGTACAACCC-3’
NOS2	5’-CCAAGCCCTCACCTACCTCC-3’	5’-CTCTGAGGGCTGACACAAGG-3’
IL6	5’-CTGCAAGAGACTTCCATCCAG-3’	5’-AGTGGTATAGACAGGTCTGTTGG-3’
ARG1	5’-AACACGGCAGTGGCTTTAAC-3’	5’-GTCAGTCCCTGGCTTATGGTT-3’
CD163	5’-CCTGGATCATCTGTCACAACA-3’	5’-TCCACACGTCCAGACCAGTC-3’
IL10	5’-CGGGAAGACAATAACTGCACCC-3’	5’-CGGTTAGCAGTATGTTGTCCAGC-3’
TGFB	5’-AGAGGTCACCCGCGTGCTAA-3’	5’-TCCCGAATGTCTGACGTATTGA-3’
CCL2	5’-TTAAAAACCTGGATCGGAACCAA-3’	5’-GCATTAGCTTCAGATTTACGGGT-3’
CCL5	5’-TGCCCACGTCAAGGAGTATTTC-3’	5’-AACCCACTTCTTCTCTGGGTTG-3’
CXCL9	5’-CCTAGTGATAAGGAATGCACGATG-3’	5’-CTAGGCAGGTTTGATCTCCGTTC-3’
CXCL12	5’-GATTGTAGCCCGGCTGAAGA-3’	5’-TTCGGGTCAATGCACACTTGT-3’
CSF1	5’-GCCTCCTGTTCTACAAGTGGAAG-3’	5’-ACTGGCAGTTCCACCTGTCTGT-3’
CX3CL1	5’-CAGTGGCTTTGCTCATCCGCTA-3’	5’-AGCCTGGTGATCCAGATGCTTC-3’
MFGE8	5’-CAACCTAGCCTCCCGTTGTT-3’	5’-CTCCTTGTCTCCACCGCTTT-3’
TIMD4	5’-GAGACACAAGAGGCCAGACAAC-3’	5’-AGCACCTTCATGTGGGAAGAT-3’
ANXA1	5’-CAGATGCCAGGGCTTTGTATG-3’	5’-GTCAGAATTGTGGTGAAGACG-3’
MMP9	5’-GCGGTCCTCACCATGAGTCC-3’	5’-TAGCGGTACAAGTATGCCTCTGC-3’
BCL2	5’-CGGTGCCACCTGTGGTCCAC-3’	5’-TCCCCCAGTTCACCCCGTCC-3’
VEGF	5’-CCATGAACTTTCTGCTGTCTT-3’	5’-ATCGCATCAGGGGCACACAG-3’
GAPDH	5’-AAGGTCATCCCAGAGCTGAA-3’	5’-CTGCTTCACCACCTTCTTGA-3’

Uncropped gels and blots for Supplementary Figures

In Supplementary Fig. 3b

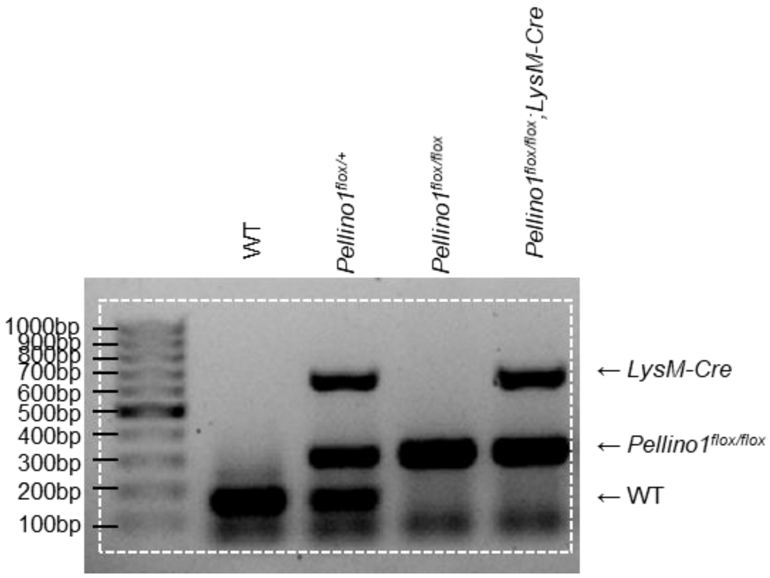

Uncropped gels and blots for Supplementary Figures

In Supplementary Fig. 13

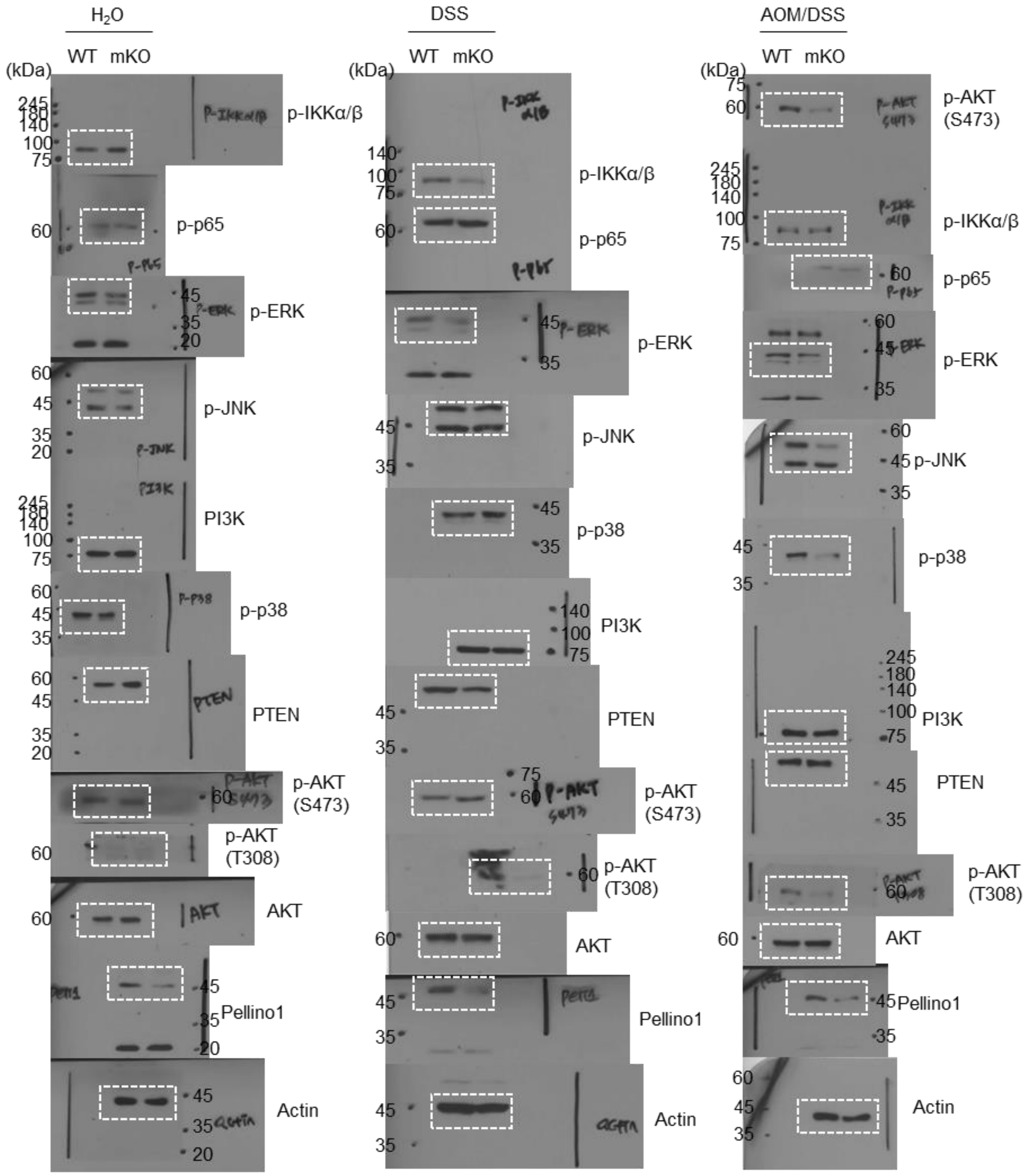

Uncropped gels and blots for Supplementary Figures

In Supplementary Fig. 14a

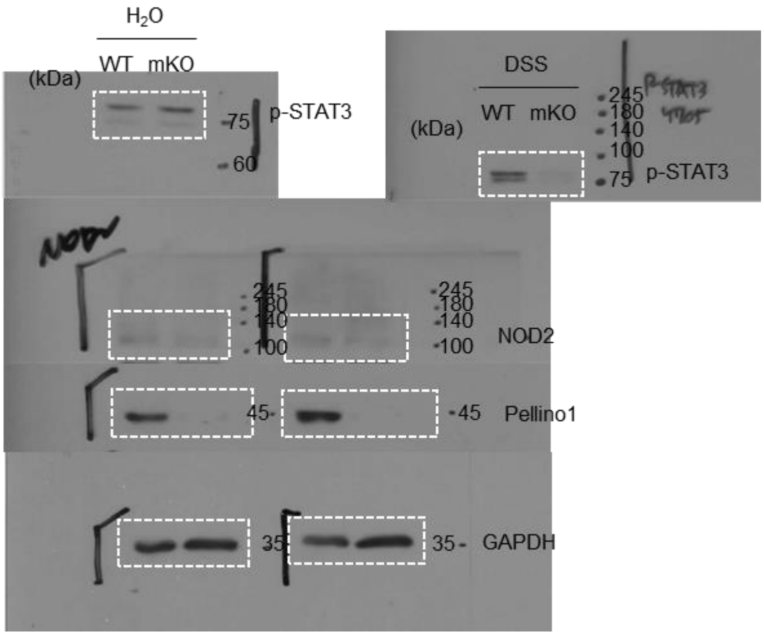

In Supplementary Fig. 14b

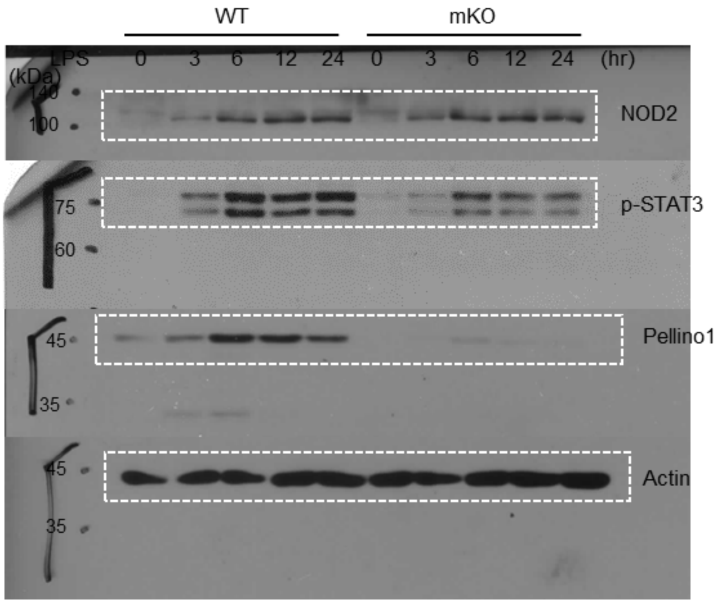

Uncropped gels and blots for Supplementary Figures

In Supplementary Fig. 14c

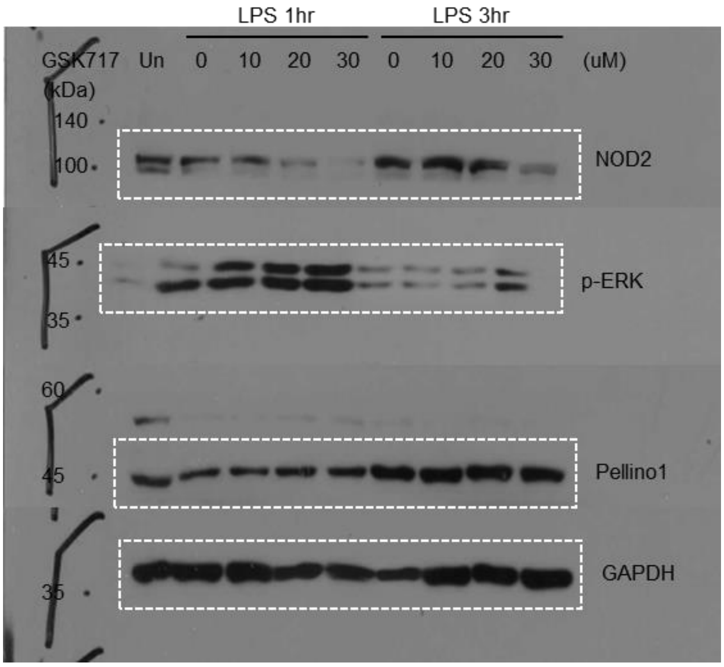

In Supplementary Fig. 14d

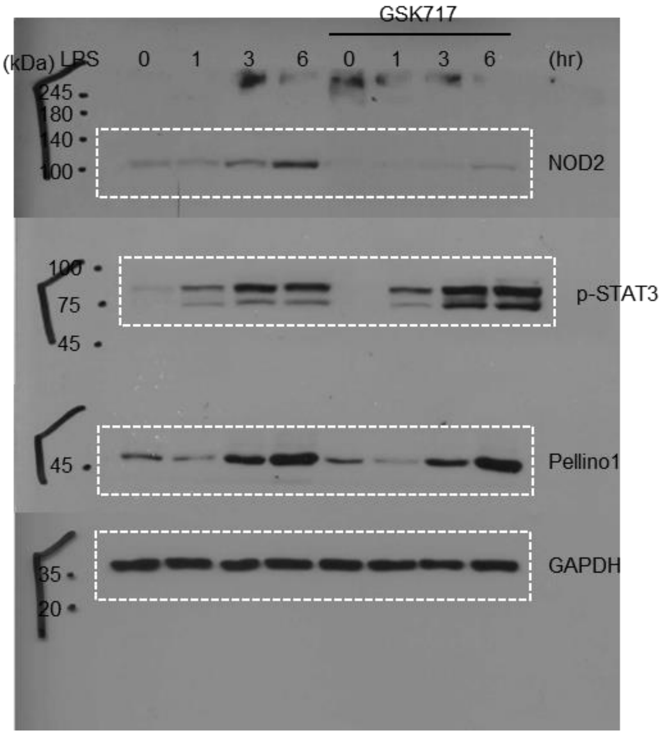

Uncropped gels and blots for Supplementary Figures

In Supplementary Fig. 15a

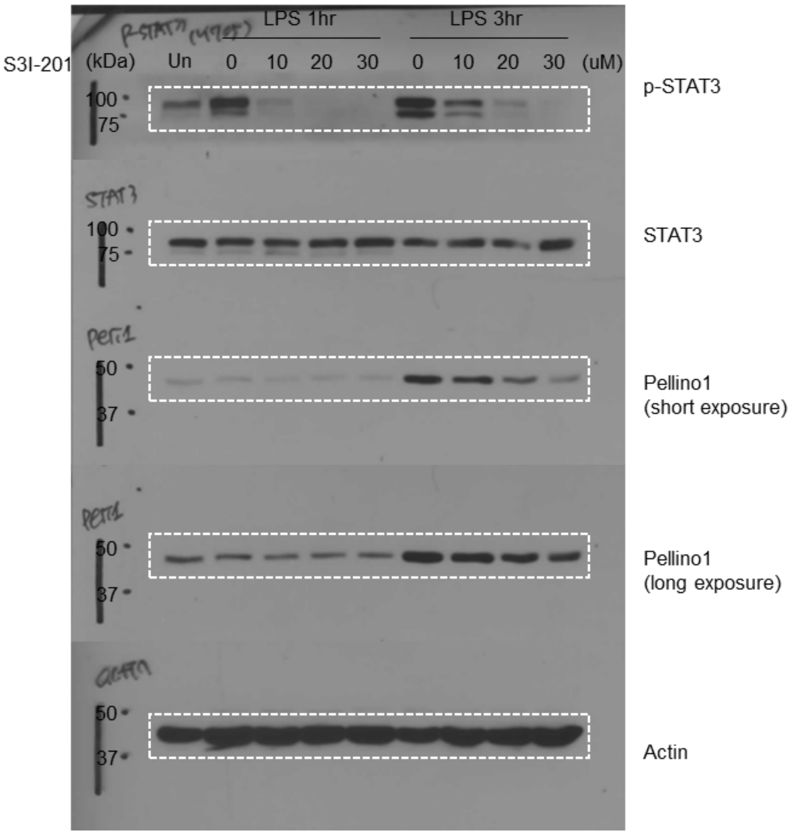

In Supplementary Fig. 15b

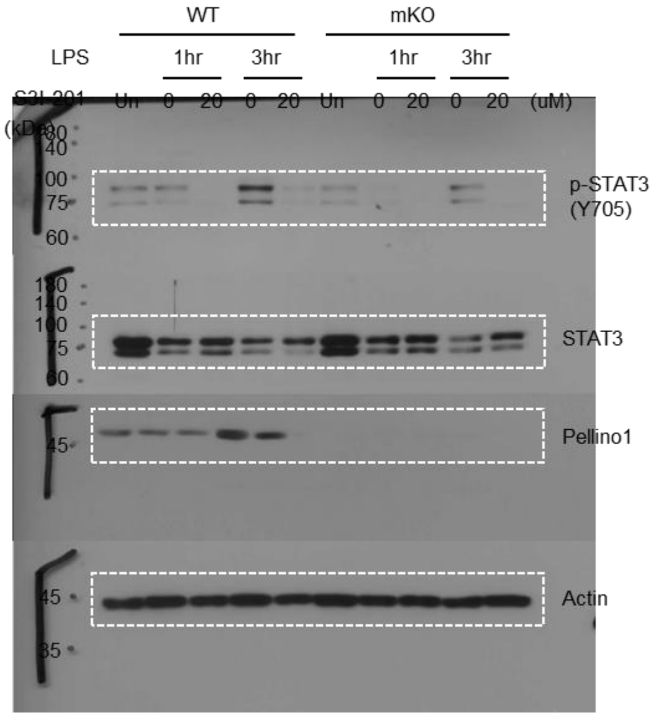

Uncropped gels and blots for Supplementary Figures

In Supplementary Fig. 16a

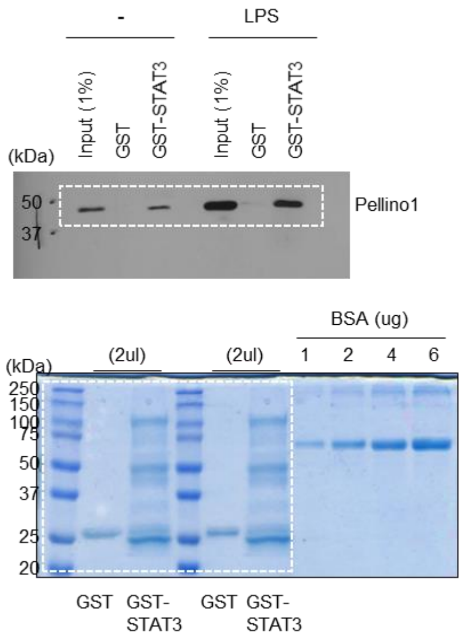

In Supplementary Fig. 16c

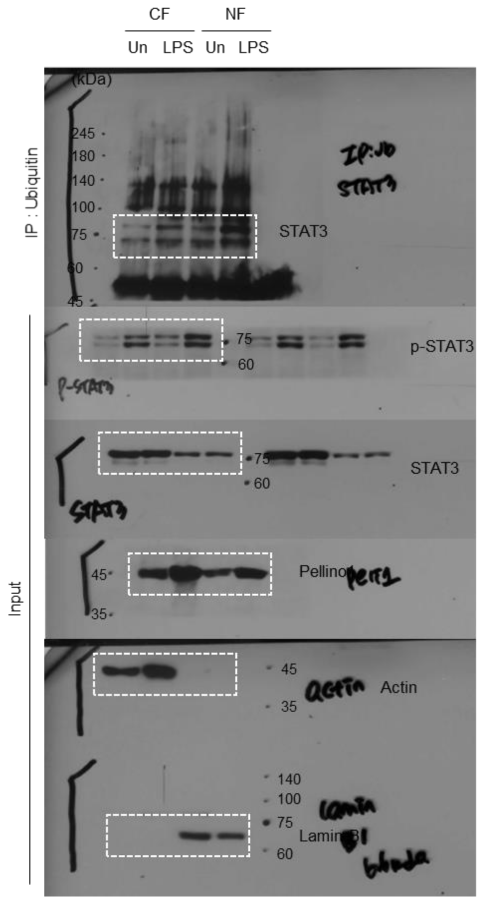

Uncropped gels and blots for Supplementary Figures

In Supplementary Fig. 16d

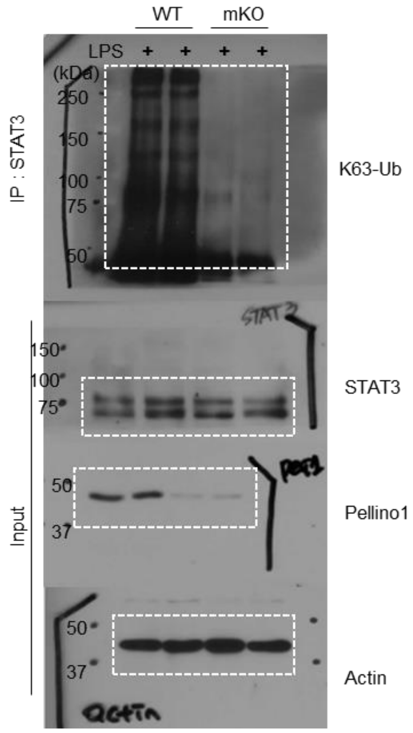

In Supplementary Fig. 16e

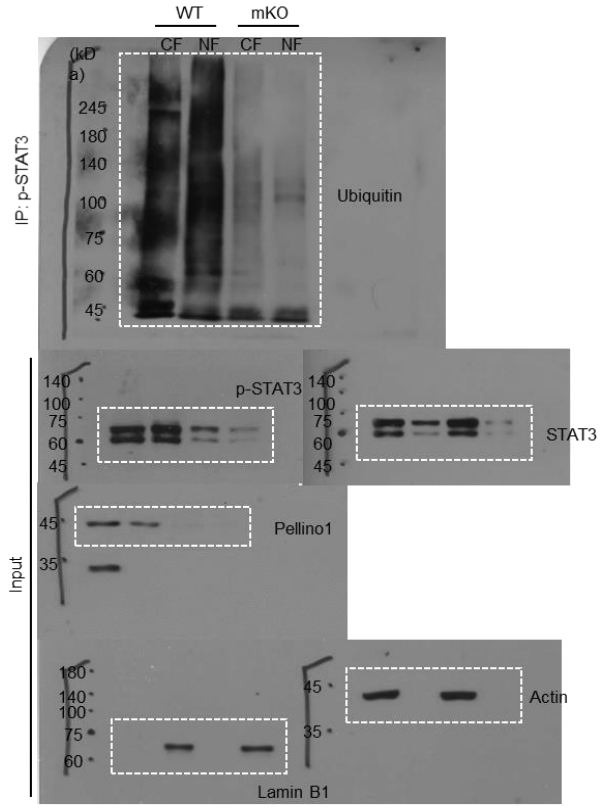

Supplement: Supplementary file 1 — Supplementary Information [file 41467_2025_56440_MOESM1_ESM.pdf]
