# Supplementary material for: The ubiquitin ligase Pellino1 targets STAT3 to regulate macrophage-mediated inflammation and tumor development
Source: Nat Commun. 2025 Feb 1;16:1256. doi: 10.1038/s41467-025-56440-6 (PMC11787384; doi:10.1038/s41467-025-56440-6)
Supplement: Supplementary file 2 — Reporting Summary [file 41467_2025_56440_MOESM2_ESM.pdf]

nature portfolio

Corresponding author(s): Chang-Woo Lee

Last updated by author(s): Dec 15, 2024

# Reporting Summary

Nature Portfolio wishes to improve the reproducibility of the work that we publish. This form provides structure for consistency and transparency in reporting. For further information on Nature Portfolio policies, see our Editorial Policies and the Editorial Policy Checklist.

## Statistics

For all statistical analyses, confirm that the following items are present in the figure legend, table legend, main text, or Methods section.

| n/a | Confirmed | |
|---|---|---|
| ☐ | ☒ | The exact sample size (*n*) for each experimental group/condition, given as a discrete number and unit of measurement |
| ☐ | ☒ | A statement on whether measurements were taken from distinct samples or whether the same sample was measured repeatedly |
| ☐ | ☒ | The statistical test(s) used AND whether they are one- or two-sided<br>*Only common tests should be described solely by name; describe more complex techniques in the Methods section.* |
| ☐ | ☒ | A description of all covariates tested |
| ☐ | ☒ | A description of any assumptions or corrections, such as tests of normality and adjustment for multiple comparisons |
| ☐ | ☒ | A full description of the statistical parameters including central tendency (e.g. means) or other basic estimates (e.g. regression coefficient) AND variation (e.g. standard deviation) or associated estimates of uncertainty (e.g. confidence intervals) |
| ☐ | ☒ | For null hypothesis testing, the test statistic (e.g. *F*, *t*, *r*) with confidence intervals, effect sizes, degrees of freedom and *P* value noted<br>*Give P values as exact values whenever suitable.* |
| ☒ | ☐ | For Bayesian analysis, information on the choice of priors and Markov chain Monte Carlo settings |
| ☒ | ☐ | For hierarchical and complex designs, identification of the appropriate level for tests and full reporting of outcomes |
| ☒ | ☐ | Estimates of effect sizes (e.g. Cohen's *d*, Pearson's *r*), indicating how they were calculated |

*Our web collection on statistics for biologists contains articles on many of the points above.*

## Software and code

Policy information about availability of computer code

| | |
|---|---|
| Data collection | QuantStudio 6 Flex Real-Time PCR (Life technologies)<br>FACSCanto II (BD biosciences)<br>MoticEasyScan Pro 6 (Motic)<br>Eclipse Ti-U (Nikon)<br>Axio Imager microscope (ZEISS)<br>Confocal laser scanning microscope LSM710 (ZEISS) |
| Data analysis | R Studio (ver. 2021.09.1 Build 372, https://www.rstudio.com/), R (ver. 4.1.2, https://www.r-project. org/), and the R packages "survminer", "survival", "multipleROC", "pROC", "RColorBrewer", "ggpubr", "dplyr", "ggplot2" and "egg" were used for statistical analyses and visualization.<br>FlowJo (version 10.7.1)<br>ImageJ (version 1.52a) (NIH, https://imagej.net/ij/)<br>AxioVision image processing software package ZEN (ZEISS, version 3.4)<br>GraphPad Prism software (version 8.0.1) (https://www.graphpad.com/features) |

For manuscripts utilizing custom algorithms or software that are central to the research but not yet described in published literature, software must be made available to editors and reviewers. We strongly encourage code deposition in a community repository (e.g. GitHub). See the Nature Portfolio guidelines for submitting code & software for further information.

## Data

Policy information about availability of data

All manuscripts must include a data availability statement. This statement should provide the following information, where applicable:

- Accession codes, unique identifiers, or web links for publicly available datasets
- A description of any restrictions on data availability
- For clinical datasets or third party data, please ensure that the statement adheres to our policy

The datasets generated and analyzed during this study are available in the public database TCGA.

Raw data for all figures are provided in the Source Data file.

## Research involving human participants, their data, or biological material

Policy information about studies with human participants or human data. See also policy information about sex, gender (identity/presentation), and sexual orientation and race, ethnicity and racism.

| | |
|---|---|
| Reporting on sex and gender | A total of 11 patients diagnosed with Crohn's disease (CD) were included in the study, comprising 2 females (18 and 24 years old, respectively), and 9 males (29, 3, 20, 20, 12, 23, 35, 15, 22 years old, respectively). Additionally, there were 11 patients diagnosed with ulcerative colitis (UC), including 6 females (23, 30, 47, 34, 34 and 48 years old), and 5 males (75, 51, 13, 27, 22 years old, respectively). The Non-IBD group consisted of 10 individuals, including 4 females (aged 64, 62, 52, 48 years old, respectively) and 6 males (58, 56, 39, 41, 61, 63 years old). |
| Reporting on race, ethnicity, or other socially relevant groupings | All patients we recruited are Korean. |
| Population characteristics | see above |
| Recruitment | The patients were consecutively and randomly collected based on diagnostic criteria, and the study is a retrospective cohort design focusing on pathological diagnoses. |
| Ethics oversight | Endoscopic biopsy specimens of patients with active IBD, including UC and CD, were collected from the pathology database of Asan Medical Center between January and December 2018. 11 cases of IBD lesions were collected, and 10 cases of normal colon tissue biopsied through health examinations were included as a control group. This retrospective study was approved by the Institutional Review Boards of the Asan Medical Center (IRB No. 2019-0701). The IRBs waived the requirement for written informed consent as it was a retrospective study. |

Note that full information on the approval of the study protocol must also be provided in the manuscript.

# Field-specific reporting

Please select the one below that is the best fit for your research. If you are not sure, read the appropriate sections before making your selection.

☒ Life sciences ☐ Behavioural & social sciences ☐ Ecological, evolutionary & environmental sciences

For a reference copy of the document with all sections, see nature.com/documents/nr-reporting-summary-flat.pdf

# Life sciences study design

All studies must disclose on these points even when the disclosure is negative.

| | |
|---|---|
| Sample size | Preliminary experiments were performed to estimate the variance for each assay and determine a sufficient sample size. Sample sizes are shown in the figure or stated in the figure legend. |
| Data exclusions | No data were excluded from analysis. |
| Replication | Animal experiments were performed with sufficient sample size, and n numbers for each experiment are stated in figure legends. All experiments were repeated at least three times and all attempts to replicate experiments were successful. |
| Randomization | For in vivo mice experiments, both age and sex-matched mice were grouped randomly according to the genotype and littermates were used where applicable. For in vitro experiments, cells are coming from the same populations and well location for cell culture were randomly arranged. |
| Blinding | Blinding was used for experiments involving pathological interpretation of histological sections and quantification of histological sections. Blinding was not used for any other experiments in this study since there was no need to avoid selection bias. |

# Reporting for specific materials, systems and methods

We require information from authors about some types of materials, experimental systems and methods used in many studies. Here, indicate whether each material, system or method listed is relevant to your study. If you are not sure if a list item applies to your research, read the appropriate section before selecting a response.

## Materials & experimental systems

| n/a | Involved in the study |
| --- | --- |
| ☐ | ☒ Antibodies |
| ☐ | ☒ Eukaryotic cell lines |
| ☒ | ☐ Palaeontology and archaeology |
| ☐ | ☒ Animals and other organisms |
| ☒ | ☐ Clinical data |
| ☒ | ☐ Dual use research of concern |
| ☒ | ☐ Plants |

## Methods

| n/a | Involved in the study |
| --- | --- |
| ☒ | ☐ ChIP-seq |
| ☐ | ☒ Flow cytometry |
| ☒ | ☐ MRI-based neuroimaging |

## Antibodies

Antibodies used

Mouse anti-PCNA (Santa Cruz Biotechnology, Cat# sc-56), 1:200
Rabbit anti-MDM2 (Santa Cruz Biotechnology, Cat# sc-812), 1:50
Rabbit anti-ERK (Santa Cruz Biotechnology, Cat# sc-94), 1:1000
Mouse anti-JNK (Santa Cruz Biotechnology, Cat# sc-7345), 1:1000
Mouse anti-GST (Santa Cruz Biotechnology, Cat# sc-138), 1:1000
Mouse anti-Pellino1 (Santa Cruz Biotechnology, Cat# sc-271065), 1:1000
Rabbit anti-GAPDH (Santa Cruz Biotechnology, Cat# sc-25778), 1:3000
Mouse anti-ubiquitin (Santa Cruz Biotechnology, Cat# sc-8017), 1:1000
Rabbit anti-iNOS (Cell Signaling Technology, Cat# 13120S), 1:200
Mouse anti-STAT3 (Cell Signaling Technology, Cat# 9139S), 1:1000
Mouse anti-Lamin B1 (Santa Cruz Biotechnology, Cat# sc-374015), 1:1000
Rabbit anti-AKT (Cell Signaling Technology, Cat# 4691S), 1:1000
Rabbit anti-p-AKT (S473) (Cell Signaling Technology, Cat# 4060S), 1:1000
Rabbit anti-p-AKT (T308) (Cell Signaling Technology, Cat# 9271S), 1:1000
Rabbit anti-p-ERK (Cell Signaling Technology, Cat# 4376S), 1:1000
Rabbit anti-p-JNK (Cell Signaling Technology, Cat# 4668S), 1:1000
Rabbit anti-p-p38 (Cell Signaling Technology, Cat# 4511S), 1:1000
Rabbit anti-p-p65 (Cell Signaling Technology, Cat# 3033S), 1:1000
Rabbit anti-p-IKKa/b (Cell Signaling Technology, Cat# 2697S), 1:1000
Rabbit anti-p-STAT3 (Y705) (Cell Signaling Technology, Cat# 9145S), 1:1000
Rabbit anti-p-STAT3 (S727) (Cell Signaling Technology, Cat# 9134S), 1:1000
Rabbit anti-PI3K (Cell Signaling Technology, Cat# 4292S), 1:1000
Rabbit anti-PTEN (Cell Signaling Technology, Cat# 9188P), 1:1000
Rabbit anti-B-Catenin (Cell Signaling Technology, Cat# 8480S), 1:200
Rabbit anti-HA-Tag (Cell Signaling Technology, Cat# 3724S), 1:1000
Mouse anti-Myc-Tag (Cell Signaling Technology, Cat# 2276S), 1:1000
Rabbit anti-K63-linkage-specific polyubiquitin (Cell Signaling Technology, Cat# 5621S), 1:1000
Rabbit anti-Flag-Tag antibody (Sigma-Aldrich, Cat# F7425), 1:1000
Rabbit anti-Actin antibody (Sigma-Aldrich, Cat# A2066), 1:3000
Rabbit anti-Ki67 antibody (GeneTex, Cat# GTX16667), 1:200
Rabbit anti-COX-2 (Abcam, Cat# ab15191), 1:1000
Rabbit anti-CD68 (Abcam, Cat# ab283654), 1:100
Rat anti-Ly6G (Abcam, Cat# Ab25377),1:100
Rat anti mouse F4/80 (Bio-Rad, Cat# MCA497GA), 1:200
Rat anti-Nod2 (26mNOD2, eBioscience, Cat# 14-5858-82) 1:1000
Rat, PE-anti-B220 (clone RA3-6B2, eBioscience, Cat# 12-0452-82)
Rat, PE-Cy7-anti-B220 (clone RA3-6B2, eBioscience, Cat# 25-0452-82)
Armenian hamster, PerCP-Cy5.5-anti-CD3 (clone 145-2C11, eBioscience, Cat# 45-0031-82)
Armenian hamster, PE-Cy7-anti-CD3 (clone 145-2C11, eBioscience, Cat# 25-0031-82)
Rat, FITC-anti-CD4 (clone RM4-5, eBioscience, Cat# 11-0042-82)
Rat, PE-Cy7-anti-CD4 (clone RM4-5, eBioscience, Cat# 25-0042-82)
Rat, PE-anti-CD8 (clone 53-6.7, eBioscience, Cat# 12-0081-82)
Rat, APC-anti-CD8 (clone 53-6.7, eBioscience, Cat# 17-0081-82)
Rat, APC-anti-CD11b (clone M1/70, eBioscience, Cat# 17-0112-82)
Rat, PE-Cy7-anti-CD11b (clone M1/70, eBioscience, Cat# 25-0112-82)
Armenian hamster, PE-Cy7-anti-CD11c (clone N418, eBioscience, Cat# 25-0114-82)
Rat, PerCP-Cy5.5-anti-CD19 (clone 1D3, eBioscience, Cat# 45-0193-82)
Rat, PerCP-Cy5.5-anti-CD45 (clone 30-F11, eBioscience, Cat# 45-0451-82)
Mouse, PE-Cy7-anti-CX3CR1 (clone SA011F11, BioLegend, Cat# 149015)
Rat, FITC-anti-Ly6G (clone 1AB, eBioscience, Cat# 11-9668-82)

Rat, APC-anti-Ly6C (clone HK1.4, eBioscience, Cat# 17-5932-82)
Rat, FITC-anti-MHC2 (clone M5/114.15,2, eBioscience, Cat# 14-5321-82)
Rat, PerCP-Cy5.5-anti-GR-1 (clone RB6-8C5, eBioscience, Cat# 45-5931-80)
Rat, PE-anti-F4/80 (clone BM8, eBioscience, Cat# 25-4801-82)
Armenian hamster, FITC-anti-CD80 (clone 16-10A1, eBioscience, Cat# 12-0801-82)
Rat, PE-Cy7-anti-CD86 (clone GL1, eBioscience, Cat# 25-0862-82)
Rat, APC-anti-CD206 (clone MR6F3, eBioscience, Cat# 17-2061-82)
Rat, FITC-anti-CD282 (TLR2) (clone 6C2, eBioscience, Cat# 11-9021-82)
Mouse, PE-anti-CD284 (TLR4) (clone HTA125, eBioscience, Cat# 12-9917-42)
Rat, PE-Cy7-anti-CD369 (Dectin-1) (clone Bg1fpj, eBioscience, Cat# 17-5859-82)
Armenian hamster, APC-anti-CD36 (clone HM36, eBioscience, Cat# 17-0362-82)

Validation

All antibodies used in this study are commercial (please see details above) and have been previously validated by the manufacturer:

Mouse anti-PCNA (Santa Cruz Biotechnology, Cat# sc-56) is validated for Western Blotting, immunoprecipitation, immunofluorescence, immunohistochemistry and flow cytometry; this antibody works for mouse, rat, human, and insect (https://www.scbt.com/ko/p/pcna-antibody-pc10).
Rabbit anti-MDM2 (Santa Cruz Biotechnology, Cat# sc-812) is validated for Western Blotting, immunoprecipitation, immunofluorescence, and solid phase ELISA; this antibody works for mouse, rat, and human (https://www.scbt.com/ko/p/mdm2-antibody-c-18#citations).
Rabbit anti-ERK (Santa Cruz Biotechnology, Cat# sc-94) is validated for Western Blotting, immunoprecipitation, immunofluorescence, immunohistochemistry, flowcytometry, and solid phase ELISA; this antibody works for mouse, rat, human, chicken, frog, and zebrafish (https://www.scbt.com/p/erk-1-antibody-k-23?requestFrom=search).
Mouse anti-JNK (Santa Cruz Biotechnology, Cat# sc-7345) is validated for Western Blotting, immunoprecipitation, immunofluorescence, immunohistochemistry, flow cytometry, and ELISA; this antibody works for mouse, rat and human (https://www.scbt.com/p/jnk-antibody-d-2?requestFrom=search).
Mouse anti-GST (Santa Cruz Biotechnology, Cat# sc-138) is validated for Western Blotting and immunoprecipitation; this antibody works for GST fusion proteins of Schistosoma japonicum origin and recombinant GST fusion proteins expressed in E.coli (specifically designed to be used in combination with GST expression vectors such as pGEX.3X and pGEX.2T) (https://www.scbt.com/ko/p/gst-antibody-b-14).
Mouse anti-Pellino1 (Santa Cruz Biotechnology, Cat# sc-271065) is validated for Western Blotting, immunoprecipitation, immunofluorescence, immunohistochemistry and ELISA; this antibody works for mouse, rat and human (https://www.scbt.com/ko/p/pellino-1-2-antibody-f-7).
Rabbit anti-GAPDH (Santa Cruz Biotechnology, Cat# sc-25778) is validated for Western Blotting, immunoprecipitation, immunofluorescence, immunohistochemistry and solid phase ELISA; this antibody works for mouse, rat and human (https://www.scbt.com/ko/p/gapdh-antibody-fl-335).
Mouse anti-ubiquitin (Santa Cruz Biotechnology, Cat# sc-8017) is validated for Western Blotting, immunoprecipitation, immunofluorescence, immunohistochemistry, flow cytometry, and ELISA; this antibody works for mouse, rat, human and drosophila (https://www.scbt.com/ko/p/ubiquitin-antibody-p4d1).
Rabbit anti-iNOS (Cell Signaling Technology, Cat# 13120S) is validated for Western Blotting, immunoprecipitation, immunofluorescence, and flow cytometry; this antibody works for mouse (https://www.cellsignal.com/products/primary-antibodies/inos-d6b6s-rabbit-mab/13120).
Mouse anti-STAT3 (Cell Signaling Technology, Cat# 9139S) is validated for Western Blotting, immunoprecipitation, IHC Leica Bond, immunohistochemistry, immunofluorescence, flow cytometry, chromatin IP, and CUT&RUN; this antibody works for mouse, rat, human and monkey (https://www.cellsignal.com/products/primary-antibodies/stat3-124h6-mouse-mab/9139).
Mouse anti-Lamin B1 (Santa Cruz Biotechnology, Cat# sc-374015) is validated for Western Blotting, immunoprecipitation, immunofluorescence, and ELISA; this antibody works for mouse, rat, and human (https://www.scbt.com/p/lamin-b1-antibody-b-10).
Rabbit anti-AKT (Cell Signaling Technology, Cat# 4691S) is validated for Western Blotting, immunoprecipitation, immunohistochemistry, immunofluorescence, and flow cytometry; this antibody works for mouse, rat, human, monkey, and D. melanogaster (https://www.cellsignal.com/products/primary-antibodies/akt-pan-c67e7-rabbit-mab/4691).
Rabbit anti-p-AKT (S473) (Cell Signaling Technology, Cat# 4060S) is validated for Western Blotting, immunoprecipitation, immunofluorescence, and flow cytometry; this antibody works for mouse, rat, human, hamster, monkey, D. melanogaster, and dog (https://www.cellsignal.com/products/primary-antibodies/phospho-akt-ser473-antibody/9271).
Rabbit anti-p-AKT (T308) (Cell Signaling Technology, Cat# 9271S) is validated for Western Blotting and immunoprecipitation; this antibody works for mouse, rat, human and monkey (https://www.cellsignal.com/products/primary-antibodies/phospho-akt-thr308-244f9-rabbit-mab/4056).
Rabbit anti-p-ERK (Cell Signaling Technology, Cat# 4376S) is validated for Western Blotting, immunoprecipitation, and immunohistochemistry; this antibody works for mouse, rat, human, hamster, monkey, mink, D. melanogaster, zebrafish, pig, and S. cerevisiae (https://www.cellsignal.com/products/primary-antibodies/phospho-p44-42-mapk-erk1-2-thr202-tyr204-20g11-rabbit-mab/4376).
Rabbit anti-p-JNK (Cell Signaling Technology, Cat# 4668S) is validated for Western Blotting, immunoprecipitation, and immunohistochemistry; this antibody works for mouse, rat, human, D. melanogaster, and S. cerevisiae (https://www.cellsignal.com/products/primary-antibodies/phospho-sapk-jnk-thr183-tyr185-81e11-rabbit-mab/4668).
Rabbit anti-p-p38 (Cell Signaling Technology, Cat# 4511S) is validated for Western Blotting, immunoprecipitation, immunohistochemistry, immunofluorescence, and flow cytometry; this antibody works for mouse, rat, human, monkey, mink, pig, and S. cerevisiae (https://www.cellsignal.com/products/primary-antibodies/phospho-p38-mapk-thr180-tyr182-d3f9-xp-rabbit-mab/4511).
Rabbit anti-p-p65 (Cell Signaling Technology, Cat# 3033S) is validated for Western Blotting, immunoprecipitation, immunofluorescence, and flow cytometry; this antibody works for mouse, rat, human, hamster, mink, and pig (https://www.cellsignal.com/products/primary-antibodies/phospho-nf-kb-p65-ser536-93h1-rabbit-mab/3033).
Rabbit anti-p-IKKa/b (Cell Signaling Technology, Cat# 2697S) is validated for Western Blotting, immunohistochemistry, and flow cytometry; this antibody works for mouse, rat, human, hamster, and mink (https://www.cellsignal.com/products/primary-antibodies/phospho-ikka-b-ser176-180-16a6-rabbit-mab/2697).
Rat anti-Nod2 (26mNOD2, eBioscience, Cat# 14-5858-82) is validated for Western Blotting; this antibody works for mouse (https://www.thermofisher.com/antibody/product/Nod2-Antibody-clone-26mNOD2-Monoclonal/14-5858-82).
Rabbit anti-p-STAT3 (Y705) (Cell Signaling Technology, Cat# 9145S) is validated for Western Blotting, immunoprecipitation, IHC Leica

Bond, immunohistochemistry, immunofluorescence, flow cytometry, chromatin IP, and chromatin IP-seq; this antibody works for mouse, rat, human and mink (https://www.cellsignal.com/products/primary-antibodies/phospho-stat3-tyr705-d3a7-xp-rabbit-mab/9145).
Rabbit anti-p-STAT3 (S727) (Cell Signaling Technology, Cat# 9134S) is validated for Western Blotting, immunoprecipitation, and chromatin IP; this antibody works for mouse, rat, and human (https://www.cellsignal.com/products/primary-antibodies/phospho-stat3-ser727-antibody/9134).
Rabbit anti-PI3K (Cell Signaling Technology, Cat# 4292S) is validated for Western Blotting and immunoprecipitation; this antibody works for mouse, rat, and human (https://www.cellsignal.com/products/primary-antibodies/pi3-kinase-p85-antibody/4292).
Rabbit anti-PTEN (Cell Signaling Technology, Cat# 9188P) is validated for Western Blotting, immunoprecipitation, IHC Leica Bond, and immunohistochemistry; this antibody works for mouse, rat, human, monkey, and dog (https://www.cellsignal.com/products/primary-antibodies/pten-d4-3-xp-rabbit-mab/9188).
Rabbit anti-B-Catenin (Cell Signaling Technology, Cat# 8480S) is validated for Western Blotting, immunoprecipitation, IHC Leica Bond, immunohistochemistry, immunofluorescence, flow cytometry, chromatin IP, chromatin IP-seq, and CUT&RUN; this antibody works for mouse, rat, human, and monkey (https://www.cellsignal.com/products/primary-antibodies/b-catenin-d10a8-xp-rabbit-mab/8480).
Rabbit anti-HA-Tag (Cell Signaling Technology, Cat# 3724S) is validated for Western Blotting, immunoprecipitation, immunohistochemistry, immunofluorescence, flow cytometry, and chromatin IP; this antibody works for mouse, rat, human, hamster, monkey, virus, mink, chicken, D. melanogaster, xenopus, zebrafish, bovine, dog, pig, S. cerevisiae, C. elegans, horse, guinea pig, and rabbit (https://www.cellsignal.com/products/primary-antibodies/ha-tag-c29f4-rabbit-mab/3724).
Mouse anti-Myc-Tag (Cell Signaling Technology, Cat# 2276S) is validated for Western Blotting, immunoprecipitation, immunohistochemistry, immunofluorescence, flow cytometry, chromatin IP; this antibody works for mouse, rat, human, hamster, monkey, virus, mink, chicken, D. melanogaster, xenopus, zebrafish, bovine, dog, pig, S. cerevisiae, C. elegans, horse, guinea pig, and rabbit (https://www.cellsignal.com/products/primary-antibodies/myc-tag-9b11-mouse-mab/2276).
Rabbit anti-K63-linkage-specific polyubiquitin (Cell Signaling Technology, Cat# 5621S) is validated for Western Blotting; this antibody works for mouse, rat, human, hamster, monkey, virus, mink, chicken, D. melanogaster, xenopus, zebrafish, bovine, dog, pig, S. cerevisiae, C. elegans, horse, guinea pig, and rabbit (https://www.cellsignal.com/products/primary-antibodies/k63-linkage-specific-polyubiquitin-d7a11-rabbit-mab/5621).
Rabbit anti-Flag-Tag antibody (Sigma-Aldrich, Cat# F7425) is validated for Western Blotting, immunoprecipitation, and indirect immunofluorescence; this antibody works for mouse, rat, human, hamster, monkey, virus, mink, chicken, D. melanogaster, xenopus, zebrafish, bovine, dog, pig, S. cerevisiae, C. elegans, horse, guinea pig, and rabbit (https://www.sigmaaldrich.com/KR/ko/product/sigma/f7425).
Rabbit anti-Actin antibody (Sigma-Aldrich, Cat# A2066) is validated for Western Blotting, immunohistochemistry, and indirect immunofluorescence; this antibody works for wide range, vertebrates, human, slime mold, amoeba, and chicken (https://www.sigmaaldrich.com/KR/ko/product/sigma/a2066).
Rabbit anti-Ki67 antibody (GeneTex, Cat# GTX16667) is validated for Western Blotting, immunofluorescence, immunohistochemistry, flow cytometry; this antibody works for mouse, rat, chicken, and pig (https://www.genetex.com/Product/Detail/Ki67-antibody-SP6/GTX16667).
Rabbit anti-COX-2 (Abcam, Cat# ab15191) is validated for Western Blotting, immunohistochemistry, and sELISA; this antibody works for mouse and human (https://www.abcam.com/en-kr/products/primary-antibodies/cox2-cyclooxygenase-2-antibody-ab15191).
Rabbit anti-CD68 (Abcam, Cat# ab283654) is validated for Western Blotting, immunofluorescence, immunoprecipitation, immunohistochemistry, and flow cytometry; this antibody works for mouse and rat (https://www.abcam.com/en-kr/products/primary-antibodies/cd68-antibody-epr23917-164-ab283654).
Rat anti-Ly6G (Abcam, Cat# Ab25377) is validated for immunohistochemistry and flow cytometry; this antibody works for mouse (https://www.abcam.com/en-kr/products/primary-antibodies/ly6g-ly6c-antibody-rb6-8c5-ab25377).
Rat anti mouse F4/80 (Bio-Rad, Cat# MCA497GA) is validated for Western Blotting, immunoprecipitation, immunohistochemistry, immunofluorescence, flow cytometry, radioimmunoassays, and immuno-electron microscopy; this antibody works for mouse (https://www.bio-rad-antibodies.com/monoclonal/mouse-f4-80-antibody-cl-a3-1-mca497.html?f=purified).
Rat, PE-anti-B220 (clone RA3-6B2, eBioscience, Cat# 12-0452-82) is validated for immunohistochemistry and flow cytometry; this antibody works for mouse and human (https://www.thermofisher.com/antibody/product/CD45R-B220-Antibody-clone-RA3-6B2-Monoclonal/12-0452-82).
Rat, PE-Cy7-anti-B220 (clone RA3-6B2, eBioscience, Cat# 25-0452-82) is validated for immunohistochemistry and flow cytometry; this antibody works for mouse and human (https://www.thermofisher.com/antibody/product/CD45R-B220-Antibody-clone-RA3-6B2-Monoclonal/25-0452-82).
Armenian hamster, PerCP-Cy5.5-anti-CD3 (clone 145-2C11, eBioscience, Cat# 45-0031-82) is validated for immunohistochemistry, functional assay, and flow cytometry; this antibody works for mouse (https://www.thermofisher.com/antibody/product/CD3e-Antibody-clone-145-2C11-Monoclonal/45-0031-82).
Armenian hamster, PE-Cy7-anti-CD3 (clone 145-2C11, eBioscience, Cat# 25-0031-82) is validated for immunohistochemistry, functional assay, and flow cytometry; this antibody works for mouse (https://www.thermofisher.com/antibody/product/CD3e-Antibody-clone-145-2C11-Monoclonal/25-0031-82).
Rat, FITC-anti-CD4 (clone RM4-5, eBioscience, Cat# 11-0042-82) is validated for immunohistochemistry, immunocytochemistry, and flow cytometry; this antibody works for mouse (https://www.thermofisher.com/antibody/product/CD4-Antibody-clone-RM4-5-Monoclonal/11-0042-82?gclid=EAIaIQobChMIuYveqaS5hAMVvGgPAh1teg52EAAYASAAEgLT4_D_BwE&ef_id=EAIaIQobChMIuYveqaS5hAMVvGgPAh1teg52EAAYASAAEgLT4_D_BwE:G:s&s_kwcid=AL!3652!3!600168785949!!!g!!!1454324556!63404918784&cid=bid_pca_frg_r01_co_cp1359_pjt0000_bid00000_0se_gaw_dy_pur_con&gad_source=1).
Rat, PE-Cy7-anti-CD4 (clone RM4-5, eBioscience, Cat# 25-0042-82) is validated for immunocytochemistry and flow cytometry; this antibody works for mouse (https://www.thermofisher.com/antibody/product/CD4-Antibody-clone-RM4-5-Monoclonal/25-0042-82?gclid=EAIaIQobChMIqsmR1aS5hAMV5tYWBR00gQJsEAAYASAAEgKN6fD_BwE&ef_id=EAIaIQobChMIqsmR1aS5hAMV5tYWBR00gQJsEAAYASAAEgKN6fD_BwE:G:s&s_kwcid=AL!3652!3!600168785949!!!g!!!1454324556!63404918784&cid=bid_pca_frg_r01_co_cp1359_pjt0000_bid00000_0se_gaw_dy_pur_con&gad_source=1).
Rat, PE-anti-CD8 (clone 53-6.7, eBioscience, Cat# 12-0081-82) is validated for Western Blotting, immunohistochemistry, immunocytochemistry, functional assay, miscellaneous pubmed, and flow cytometry; this antibody works for mouse (https://www.thermofisher.com/antibody/product/CD8a-Antibody-clone-53-6-7-Monoclonal/12-0081-82).
Rat, APC-anti-CD8 (clone 53-6.7, eBioscience, Cat# 17-0081-82) is validated for immunohistochemistry, immunocytochemistry, functional assay, miscellaneous pubmed, and flow cytometry; this antibody works for mouse (https://www.thermofisher.com/antibody/product/CD8a-Antibody-clone-53-6-7-Monoclonal/17-0081-82).

Rat, APC-anti-CD11b (clone M1/70, eBioscience, Cat# 17-0112-82) is validated for immunohistochemistry, immunocytochemistry, in situ PLA, miscellaneous pubmed, and flow cytometry; this antibody works for mouse (https://www.thermofisher.com/antibody/product/CD11b-Antibody-clone-M1-70-Monoclonal/17-0112-82?gclid=EAIaIQobChMI0r29pqW5hAMVWQl7Bx0xNAHnEAAYASAAEgJS2vD_BwE&ef_id=EAIaIQobChMI0r29pqW5hAMVWQl7Bx0xNAHnEAAYASAAEgJS2vD_BwE:G:s&s_kwcid=AL!3652!3!600168785949!!!g!!!1454324556!63404918784&cid=bid_pca_frg_r01_co_cp1359_pjt0000_bid00000_0se_gaw_dy_pur_con&gad_source=1).
Rat, PE-Cy7-anti-CD11b (clone M1/70, eBioscience, Cat# 25-0112-82) is validated for miscellaneous pubmed and flow cytometry; this antibody works for mouse (https://www.thermofisher.com/antibody/product/CD11b-Antibody-clone-M1-70-Monoclonal/25-0112-82).
Armenian hamster, PE-Cy7-anti-CD11c (clone N418, eBioscience, Cat# 25-0114-82) is validated for immunohistochemistry and flow cytometry; this antibody works for mouse (https://www.thermofisher.com/antibody/product/CD11c-Antibody-clone-N418-Monoclonal/25-0114-82).
Rat, PerCP-Cy5.5-anti-CD19 (clone 1D3, eBioscience, Cat# 45-0193-82) is validated for immunocytochemistry and flow cytometry; this antibody works for mouse (https://www.thermofisher.com/antibody/product/CD19-Antibody-clone-eBio1D3-1D3-Monoclonal/45-0193-82).
Rat, PerCP-Cy5.5-anti-CD45 (clone 30-F11, eBioscience, Cat# 45-0451-82) is validated for flow cytometry; this antibody works for mouse (https://www.thermofisher.com/antibody/product/CD45-Antibody-clone-30-F11-Monoclonal/45-0451-82).
Mouse, PE-Cy7-anti-CX3CR1 (clone SA011F11, BioLegend, Cat# 149015) is validated for flow cytometry; this antibody works for mouse (https://www.biolegend.com/en-ie/products/pe-cyanine7-anti-mouse-cx3cr1-antibody-11909?GroupID=BLG13324).
Rat, FITC-anti-Ly6G (clone 1AB, eBioscience, Cat# 11-9668-82) is validated for immunohistochemistry, immunocytochemistry, and flow cytometry; this antibody works for mouse (https://www.thermofisher.com/antibody/product/Ly-6G-Antibody-clone-1A8-Ly6g-Monoclonal/11-9668-82).
Rat, APC-anti-Ly6C (clone HK1.4, eBioscience, Cat# 17-5932-82) is validated for flow cytometry; this antibody works for mouse (https://www.thermofisher.com/antibody/product/Ly-6C-Antibody-clone-HK1-4-Monoclonal/17-5932-82?gclid=EAIaIQobChMI6MHrn6a5hAMVV9EWBR0rnQGlEAAYASAAEgKGivD_BwE&ef_id=EAIaIQobChMI6MHrn6a5hAMVV9EWBR0rnQGlEAAYASAAEgKGivD_BwE:G:s&s_kwcid=AL!3652!3!600168785949!!!g!!!1454324556!63404918784&cid=bid_pca_frg_r01_co_cp1359_pjt0000_bid00000_0se_gaw_dy_pur_con&gad_source=1).
Rat, FITC-anti-MHC2 (clone M5/114.15,2, eBioscience, Cat# 14-5321-82) is validated for Western Blotting, immunohistochemistry, immunocytochemistry, flow cytometry, ELISA, immunoprecipitation, neutralization, functional assay, inhibition assays, blocking assay, In vitro assay, and miscellaneous pubmed; this antibody works for mouse (https://www.thermofisher.com/antibody/product/MHC-Class-II-I-A-I-E-Antibody-clone-M5-114-15-2-Monoclonal/14-5321-82?gclid=EAIaIQobChMIz-H4pqa5hAMVrQ57Bx3-JQElEAAYASAAEgLcYfD_BwE&ef_id=EAIaIQobChMIz-H4pqa5hAMVrQ57Bx3-JQElEAAYASAAEgLcYfD_BwE:G:s&s_kwcid=AL!3652!3!600168785949!!!g!!!1454324556!63404918784&cid=bid_pca_frg_r01_co_cp1359_pjt0000_bid00000_0se_gaw_dy_pur_con&gad_source=1).
Rat, PerCP-Cy5.5-anti-GR-1 (clone RB6-8C5, eBioscience, Cat# 45-5931-80) is validated for flow cytometry and functional assay; this antibody works for mouse (https://www.thermofisher.com/antibody/product/Ly-6G-Ly-6C-Antibody-clone-RB6-8C5-Monoclonal/45-5931-80).
Rat, PE-anti-F4/80 (clone BM8, eBioscience, Cat# 25-4801-82) is validated for immunohistochemistry and flow cytometry; this antibody works for mouse (https://www.thermofisher.com/antibody/product/F4-80-Antibody-clone-BM8-Monoclonal/25-4801-82?gclid=EAIaIQobChMIqYiKt6a5hAMVDQt7Bx2CxwZEEAAYASAAEgKAVPD_BwE&ef_id=EAIaIQobChMIqYiKt6a5hAMVDQt7Bx2CxwZEEAAYASAAEgKAVPD_BwE:G:s&s_kwcid=AL!3652!3!600168785949!!!g!!!1454324556!63404918784&cid=bid_pca_frg_r01_co_cp1359_pjt0000_bid00000_0se_gaw_dy_pur_con&gad_source=1).
Armenian hamster, FITC-anti-CD80 (clone 16-10A1, eBioscience, Cat# 12-0801-82) is validated for immunohistochemistry, immunocytochemistry, In vitro assay, and flow cytometry; this antibody works for mouse, dog, and pig (https://www.thermofisher.com/antibody/product/CD80-B7-1-Antibody-clone-16-10A1-Monoclonal/12-0801-82?gclid=EAIaIQobChMI9ca9vqa5hAMVGNpMAh0BZQDgEAAYAiAAEgL8qPD_BwE&ef_id=EAIaIQobChMI9ca9vqa5hAMVGNpMAh0BZQDgEAAYAiAAEgL8qPD_BwE:G:s&s_kwcid=AL!3652!3!600168785949!!!g!!!1454324556!63404918784&cid=bid_pca_frg_r01_co_cp1359_pjt0000_bid00000_0se_gaw_dy_pur_con&gad_source=1).
Rat, PE-Cy7-anti-CD86 (clone GL1, eBioscience, Cat# 25-0862-82) is validated for flow cytometry; this antibody works for mouse (https://www.thermofisher.com/antibody/product/CD86-B7-2-Antibody-clone-GL1-Monoclonal/25-0862-82).
Rat, APC-anti-CD206 (clone MR6F3, eBioscience, Cat# 17-2061-82) is validated for flow cytometry; this antibody works for mouse (https://www.thermofisher.com/antibody/product/CD206-MMR-Antibody-clone-MR6F3-Monoclonal/17-2061-82?gclid=EAIaIQobChMI146BzKa5hAMVnf9MAh0XEwdSEAAYASAAEgLSWfD_BwE&ef_id=EAIaIQobChMI146BzKa5hAMVnf9MAh0XEwdSEAAYASAAEgLSWfD_BwE:G:s&s_kwcid=AL!3652!3!600168785949!!!g!!!1454324556!63404918784&cid=bid_pca_frg_r01_co_cp1359_pjt0000_bid00000_0se_gaw_dy_pur_con&gad_source=1).
Rat, FITC-anti-CD282 (TLR2) (clone 6C2, eBioscience, Cat# 11-9021-82) is validated for immunohistochemistry, immunocytochemistry, and flow cytometry; this antibody works for mouse (https://www.thermofisher.com/antibody/product/CD282-TLR2-Antibody-clone-6C2-Monoclonal/11-9021-82).
Mouse, PE-anti-CD284 (TLR4) (clone HTA125, eBioscience, Cat# 12-9917-42) is validated for immunohistochemistry, functional assay, and flow cytometry; this antibody works for human (https://www.thermofisher.com/antibody/product/CD284-TLR4-Antibody-clone-HTA125-Monoclonal/12-9917-42?imageId=120919).
Rat, PE-Cy7-anti-CD369 (Dectin-1) (clone Bg1fpj, eBioscience, Cat# 17-5859-82) is validated for flow cytometry; this antibody works for mouse (https://www.thermofisher.com/antibody/product/CD369-Clec7a-Dectin-1-Antibody-clone-bg1fpj-Monoclonal/17-5859-82?gclid=EAIaIQobChMIxqvO8Ka5hAMVBdcWBR1X3wDXEAAYASAAEgJWw_D_BwE&ef_id=EAIaIQobChMIxqvO8Ka5hAMVBdcWBR1X3wDXEAAYASAAEgJWw_D_BwE:G:s&s_kwcid=AL!3652!3!600168785949!!!g!!!1454324556!63404918784&cid=bid_pca_frg_r01_co_cp1359_pjt0000_bid00000_0se_gaw_dy_pur_con&gad_source=1).
Armenian hamster, APC-anti-CD36 (clone HM36, eBioscience, Cat# 17-0362-82) is validated for flow cytometry; this antibody works for mouse (https://www.thermofisher.com/antibody/product/CD36-Antibody-clone-HM36-Monoclonal/17-0362-82).

## Eukaryotic cell lines

Policy information about cell lines and Sex and Gender in Research

| | |
|---|---|
| Cell line source(s) | Murine cell line RAW 264.7 was purchased from ATCC (#TIB-71). |

| | |
|---|---|
| Cell line source(s) | Murine colon adenocarcinoma cell line MC38 was purchased from Kerafast (#ENH204-FP). Human cell line HEK293T was purchased from ATCC (#CRL-3216). |
| Authentication | None of the cell lines were authenticated. |
| Mycoplasma contamination | Cell lines were not tested for mycoplasma contamination. |
| Commonly misidentified lines (See ICLAC register) | No commonly misidentified cell lines were used in the study |

# Animals and other research organisms

Policy information about studies involving animals; ARRIVE guidelines recommended for reporting animal research, and Sex and Gender in Research

| | |
|---|---|
| Laboratory animals | Pellino1 WT mice on C57BL/6J background (Pellino1 flox/flox): ages 6-8 weeks old, age and sex matched, males and females used. Pellino1-mKO mice on C57BL/6J background (Pellino1 flox/flox;LysM-Cre): ages 6-8 weeks old, age and sex matched, males and females used.<br><br>Mice were maintained under controlled temperature (23°C) and humidity (40 – 60%) conditions with free access to food (normal chow diet; #5053, LabDiet) and water, except for mice used in certain experiments described in paper. |
| Wild animals | No wild animals were used in the study |
| Reporting on sex | Both male and female mice aged 6-8 weeks were used at the time of experiments unless indicated otherwise. Sample sizes for mouse experiments were empirically determined, and mice were randomly allocated to the control or experimental groups. Animal studies were conducted in a gender- and age-matched manner using littermates for each experiment.<br><br>The DSS-induced colitis and AOM/DSS-induced CAC models were created using male mice, as female mice are known to be partially protected from DSS-induced colitis due to the sex hormone estradiol. |
| Field-collected samples | No field collected samples were used in the study. |
| Ethics oversight | All animal experiments were conducted in accordance with Institutional Animal Care and Use Committee guidelines (IACUC 2020–11–03–1, IACUC 2021-11-27-3, IACUC 2022-10-34-1, IACUC 2023-01-08-1, IACUC 2023-10-16-1, and IACUC 2024-03-60-1) of Sungkyunkwan University School of Medicine (SUSM). The SUSM is accredited by the Association for Assessment and Accreditation of Laboratory Animal Care International (AAALAC International) in compliance with the Institute of Laboratory Animal Resources (ILAR) guidelines. |

Note that full information on the approval of the study protocol must also be provided in the manuscript.

# Plants

| | |
|---|---|
| Seed stocks | N/A |
| Novel plant genotypes | N/A |
| Authentication | N/A |

# Flow Cytometry

## Plots

Confirm that:

- [ ] The axis labels state the marker and fluorochrome used (e.g. CD4-FITC).
- [x] The axis scales are clearly visible. Include numbers along axes only for bottom left plot of group (a 'group' is an analysis of identical markers).
- [x] All plots are contour plots with outliers or pseudocolor plots.
- [x] A numerical value for number of cells or percentage (with statistics) is provided.

## Methodology

| | |
|---|---|
| Sample preparation | Colon tissues were chopped and digested using 1 mg/mL collagenase D and 0.2 mg/mL DNase I at 37°C for 30 minutes. The |

| | |
|---|---|
| Sample preparation | samples were then passed through 70-µm cell strainers, resuspended in 44% Percoll, and layered onto of 67% Percoll. This procedure was followed by centrifugation at 800 g for 20 minutes at room temperature. The cells located at the interface between the 67% and 44% Percoll layers were collected. The prepared cells were stained with antibodies.<br><br>For phagocytosis assay, BMDMs were stimulated with 100 ng/mL LPS for 6 hours. MC38 cells, labeled with 1uM CFSE, were co-cultured with BMDMs for 2 hours. Subsequently, cells were stained with anti-F4/80.<br><br>Immune cells were obtained by extracting cells from bone marrow or lysing spleen and lymph nodes, followed by filtration using 70-µm cell strainers and removal of red blood cells using RBC lysis buffer. Antibodies were then used to stain the obtained immune cells. |
| Instrument | BD FACS Canto II |
| Software | FlowJo (version 10.7.1) |
| Cell population abundance | In general, a stopping gate of 10,000 to 50,000 was used on total CD45+ cells. Final gated populations generally ranged in 500 to 10,000 cells, and samples with less than 50 events in the final gated population were excluded. |
| Gating strategy | Cells were initially gated based on FSC-A/SSC-A to exclude debris. Subsequently, single cells were gated according to the boundaries between negatively and positively labeled cell populations. Unlabeled cells and single-labeled cells with each antibody were used to set boundaries. |

☒ Tick this box to confirm that a figure exemplifying the gating strategy is provided in the Supplementary Information.
